# A distinct mechanism of epigenetic reprogramming silences *PAX2* and initiates endometrial carcinogenesis

**DOI:** 10.1172/JCI190989

**Published:** 2025-08-15

**Authors:** Subhransu S. Sahoo, Susmita G. Ramanand, Ileana C. Cuevas, Yunpeng Gao, Sora Lee, Ahmed Abbas, Xunzhi Zhang, Ashwani Kumar, Prasad Koduru, Sambit Roy, Russell R. Broaddus, Victoria L. Bae-Jump, Andrew B. Gladden, Jayanthi Lea, Elena Lucas, Chao Xing, Akio Kobayashi, Ram S. Mani, Diego H. Castrillon

**Affiliations:** 1 Department of Pathology,; 2 Department of Internal Medicine,; 3 McDermott Center of Human Growth and Development, and; 4 Harold C. Simmons Comprehensive Cancer Center, UT Southwestern, Dallas, Texas, USA.; 5 Department of Pathology and Lab Medicine, and; 6 Division of Gynecologic Oncology, University of North Carolina School of Medicine, Chapel Hill, North Carolina, USA.; 7 Division of Gynecologic Oncology, Department of Obstetrics and Gynecology, and; 8 Department of Obstetrics and Gynecology, UT Southwestern, Dallas, Texas, USA.; 9 Department of Kidney Development, Institute of Molecular Embryology and Genetics, Kumamoto University, Kumamoto, Japan.; 10 Department of Urology, UT Southwestern, Dallas, Texas, USA.

**Keywords:** Oncology, Reproductive biology, Mouse models, Obstetrics/gynecology, Tumor suppressors

## Abstract

Functional inactivation of tumor suppressor genes drives cancer initiation, progression, and treatment responses. Most tumor suppressor genes are inactivated through 1 of 2 well-characterized mechanisms: DNA-level mutations, such as point mutations or deletions, and promoter DNA hypermethylation. Here, we report a distinct third mechanism of tumor suppressor inactivation based on alterations to the histone rather than DNA code. We demonstrated that *PAX2* is an endometrial tumor suppressor recurrently inactivated by a distinct epigenetic reprogramming event in more than 80% of human endometrial cancers. Integrative transcriptomic, epigenomic, 3D genomic, and machine learning analyses showed that *PAX2* transcriptional downregulation is associated with replacement of open/active chromatin features (H3K27ac/H3K4me3) with inaccessible/repressive chromatin features (H3K27me3) in a framework dictated by 3D genome organization. The spread of the repressive H3K27me3 signal resembled a pearl necklace, with its length modulated by cohesin loops, thereby preventing transcriptional dysregulation of neighboring genes. This mechanism, involving the loss of a promoter-proximal superenhancer, was shown to underlie transcriptional silencing of *PAX2* in human endometrial cancers. Mouse and human preclinical models established *PAX2* as a potent endometrial tumor suppressor. Functionally, PAX2 loss promoted endometrial carcinogenesis by rewiring the transcriptional landscape via global enhancer reprogramming. The discovery that most endometrial cancers originate from a recurring epigenetic alteration carries profound implications for their diagnosis and treatment.

## Abstract 2

<span>We demonstrate that PAX2 is silenced by a highly recurrent epigenetic mechanism to initiate most endometrial cancers, establishing what we believe to be a novel mechanism of tumor suppressor inactivation.</span>

## Graphical abstract

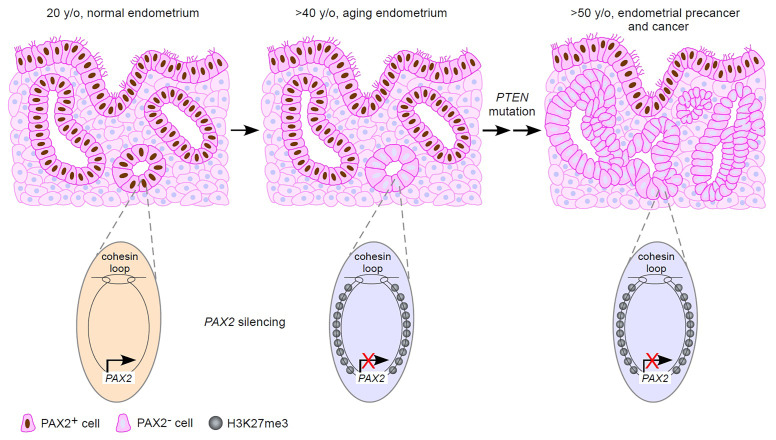

## Introduction

Endometrial cancer (EC) accounts for 7% of all cancers and is the fourth most common cancer in women, with more than 68,000 cases anticipated per year in the United States. In 2024, for the first time, EC deaths exceeded those of ovarian cancer (13,250 vs. 12,750) (1), underscoring EC’s significance as a women’s health issue. In contrast to declining incidence and improved survival for most cancers, EC incidence and mortality have been increasing over the past 40 years, by approximately 1% each year (1, 2). Age is the most significant risk factor, with EC incidence peaking in the seventh decade. Increasing life expectancy and other risk factors such as obesity contribute to EC’s rising incidence, but other poorly understood environmental and genetic factors are also at play (3–5).

EC arises from epithelial cells through a preinvasive histologic precursor termed endometrioid intraepithelial neoplasia (EIN), which progresses to invasive and lethal endometrioid adenocarcinoma (6). The EC landscape of somatically acquired driver mutations has been defined by next-generation sequencing (NGS) (5–8). *PTEN* is the most mutated gene (~50% of EC) with *PIK3R1* and *PIK3CA* mutations also frequent, underscoring a central role of PI3K/PTEN signaling (7). Epigenetic transcriptional silencing through hypermethylation of a *MLH1* promoter CpG island is another mechanism of tumor suppressor inactivation in some EC (9). CpG methylation is detectable at the genomic level with single-base resolution by methylation NGS, which has established CpG island hypermethylation as a common mechanism underlying tumor suppressor inactivation (e.g., *APC*, *BRCA1*, *CDKN2A*, *MGMT*, and *VHL*) (10, 11). However, NGS-based approaches would miss other types of nonmutational locus-specific epigenomic reprogramming events that may be critical or even initiating molecular driver events in cancer (11, 12).

Paired box gene 2 (PAX2) is one of 9 mammalian paired box DNA-binding transcription factors (TFs) (PAX1–9) with diverse roles in cell proliferation, lineage determination, organogenesis, and cancer. PAX2 is expressed in and is required for the development of the embryonic kidney and female reproductive tract; PAX2-knockout mice fail to develop kidneys or a uterus (13). Per reports in the clinical literature, PAX2 expression in endometrial glands persists into adulthood, but loss of PAX2 protein characterizes 80% of EIN and EC (8, 14–18). To date, there has been no adequate explanation of the mechanistic basis of PAX2 protein loss in the endometrium or its functional consequences.

Although it has been suggested that abnormal promoter methylation underlies PAX2 protein loss (19), this hypothesis lacks supporting evidence (20), and there have not been investigations establishing PAX2 as a functionally significant in vivo tumor suppressor. Here, we demonstrate through complementary approaches employing human specimens, cell lines, patient-derived xenografts (PDXs), and a conditional *Pax2* mouse EC model that *PAX2* inactivation is an early (initiating) event caused by a specific nonmutational epigenetic reprogramming event unrelated to abnormal methylation but instead related to the replacement of open/active (H3K27ac and H3K4me3) with inaccessible/repressive (H3K27me3) chromatin features. These epigenetic processes occur within the confines of a cohesin-mediated 3D genomic architecture, thereby preventing transcriptional dysregulation of neighboring genes and limiting *PAX2* silencing as a focal epigenetic event. *PAX2* inactivation confers a competitive growth advantage driving endometrial cell outgrowth by reprogramming endometrial transcription via the commissioning and decommissioning of thousands of enhancers. These results establish *PAX2* epigenetic silencing as a specific, early, and highly recurring molecular driver event in EC, revealing what we believe to be a new paradigm for cancer-driving gene-level epigenetic alterations and creating directions for EC research.

## Results

### Emergence of PAX2-null clones in endometrium is age dependent, and PAX2 loss characterizes 80% of EC primary tumors and cell lines.

**Figure 1:**
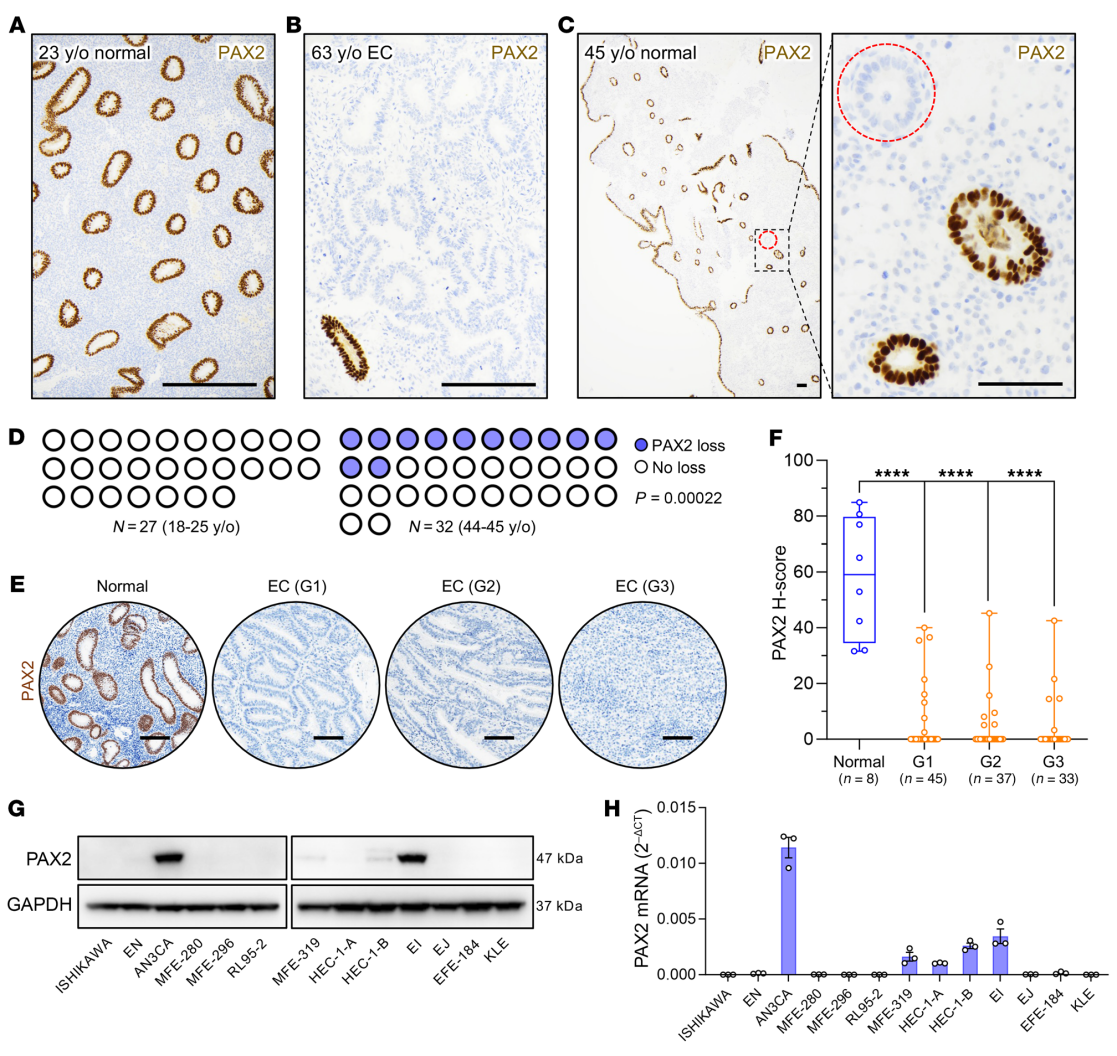
Emergence of PAX2-deficient clones in endometrial epithelium is age dependent and associated with carcinogenesis. (**A**) PAX2 immunolocalization of endometrial tissue section from younger (18–25 y/o) patient group. No PAX2-deficient clones were detected across entire specimen; representative region shown. Scale bar: 200 μm. (**B**) EC from 63 y/o patient showing complete loss of PAX2, which occurs in 80% of EC. Residual normal (non-neoplastic) gland in lower left corner underscores striking and complete loss of PAX2 expression in EC. Scale bar: 200 μm. (**C**) Endometrial tissue section from older (44–45 y/o) patient group. Dashed red circle highlights single gland in entire specimen with PAX2 loss; only portion of section shown. Right panel, magnification of boxed area showing complete (clonal) loss in all cells of the gland. Scale bars: 100 μm. (**D**) Parts of whole plots show cases with PAX2 protein loss among younger (*n* = 27) and older (*n* = 32) patients. *P* value per 2-sided Fisher’s exact test. (**E**) PAX2 expression in normal proliferative endometrium and loss in most (>80%) ECs of grades 1–3. G1, grade 1; G2, grade 2; G3, grade 3. Scale bars: 100 μm. (**F**) Box-and-whisker plots of PAX2 protein expression levels per H-scores in normal endometrium (*n* = 8) and ECs (grade 1, *n* = 45; grade 2, *n* = 37; grade 3, *n* = 33). ****P < 0.0001 per Dunnett’s multiple-comparison test. (**G**) Western blot analysis of human EC cell line panel (*n* = 13) with same PAX2 monoclonal antibody used for immunolocalization. Only 2/13 lines (AN3CA and EI) expressed normal levels of PAX2, consistent with the observed loss in approximately 80% of primary EC. (**H**) *PAX2* mRNA expression levels across human EC lines per qPCR (*n* = 3, mean ± SEM).

PAX2 is expressed in Müllerian duct epithelium during embryogenesis (13), and strong expression persists in endometrial gland epithelium into adulthood, without expression in other uterine cell types (Figure 1A). PAX2 protein loss has been reported in more than 80% of EC (Figure 1B) (8). Minute PAX2-deficient clones (defined as loss of protein expression in all cells of ≥1 endometrial gland in cross section) can be detected in some normal endometria, suggesting an early neoplastic event (14). To investigate the association of this phenomenon with age, we assessed PAX2 expression in endometria of women aged 18–25 and 44–45 years old (y/o). These age groups were chosen because, in younger or older individuals, the endometrium is underdeveloped/atrophic due to low estrogen, making this the widest age range permitting meaningful assessments. PAX2 loss was not identified in 27 patients in the 18–25 y/o group. In contrast, clonal PAX2 loss was much more frequent (12/32 cases) in the 44–45 y/o group (Figure 1C) (8, 14, 15). The difference between the age groups was statistically significant (Figure 1D; *P* = 0.00022), establishing that the emergence of PAX2-null clones is age dependent, as expected for a molecular event initiating endometrial neoplasia.

The absence of PAX2 protein in more than 80% of primary EC was consistent with previous findings (16). Notably, there was no difference among grade 1 (*n* = 45), 2 (*n* = 37), or 3 (*n* = 33) EC. The similar incidence of PAX2 loss in high-grade EC relative to low-grade EC and EIN indicates that PAX2 loss is an early driver event in EC development that usually occurs prior to the formation of a noninvasive precancer (Figure 1, E and F). Western blot analysis of PAX2 of a panel of 13 human EC cell lines revealed that PAX2 protein was undetectable in 9/13 lines and barely detectable in 2/13 (MFE-319 and HEC-1-B) (Figure 1G). Quantitative PCR (qPCR) results correlated with these findings, suggesting that transcriptional silencing may account for PAX2 protein loss in EC (Figure 1H). The absence of PAX2 protein in 11/13 (85%) EC lines validated this cell line panel as an experimental system to investigate the origins and functional consequences of PAX2 loss.

### Suppression of cell proliferation by reexpression in PAX2-deficient EC lines.

Loss of PAX2 protein in EIN and EC has been documented in clinical pathology studies, but this does not establish a causal link to carcinogenesis. To investigate this, we engineered an inducible lentiviral Tet-On system that permits precise control of *PAX2* expression (Supplemental Figure 1, A and B; supplemental material available online with this article; https://doi.org/10.1172/JCI190989DS1). Doxycycline (DOX) treatment resulted in *PAX2* reexpression in PAX2-deficient Ishikawa EC cells (Supplemental Figure 1, C and D). In vitro assays showed that reexpression resulted in significant suppression of proliferation (Supplemental Figure 1E) and reduced colony formation (Supplemental Figure 1, F and G). In vivo xenograft assays comparing empty and *PAX2* vectors (with DOX-induced expression via drinking water) demonstrated that *PAX2* reexpression resulted in tumor xenografts with slower growth and reduced size (Supplemental Figure 1, H–J). Western blot analysis and immunolocalization at euthanasia confirmed sustained PAX2 protein expression at the end of the experiment (Supplemental Figure 1, K and L). Similar experiments conducted using another PAX2-deficient EC line, HEC-1-A, yielded comparable results (Supplemental Figure 1, M–V).

### Promotion of cell proliferation by PAX2 knockdown in a PAX2-expressing line.

We investigated the effects of *PAX2* knockdown (KD) using a lentiviral construct in the EC line AN3CA (Supplemental Figure 2, A–C), which expresses PAX2 (Figure 1, G and H). In contrast to *PAX2* reexpression in Ishikawa cells, *PAX2* KD resulted in increased cell proliferation (Supplemental Figure 2D) and accelerated wound closure in 2D wound assays (Supplemental Figure 2, E and F). Cell cycle analysis showed that *PAX2* KD affected cell cycle progression, with increased numbers of cells in the S and G2/M phases and a concomitant decrease in cells in the G0/G1 phase (Supplemental Figure 2, G and H). Xenograft assays comparing scrambled shRNA control with *PAX2* shRNA showed that KD resulted in more rapid tumor growth and larger tumors (Supplemental Figure 2, I–K). Western blot analysis of tumor lysates at the end of the experiment confirmed stable *PAX2* KD (Supplemental Figure 2L). Taken together, these complementary sets of experiments provide preliminary evidence for *PAX2* as a significant endometrial tumor suppressor, whose inactivation promotes EC cell mitotic proliferation.

### Evidence against intragenic rearrangements and for PAX2 transcriptional silencing as the underlying mechanism for PAX2 inactivation.

**Figure 2:**
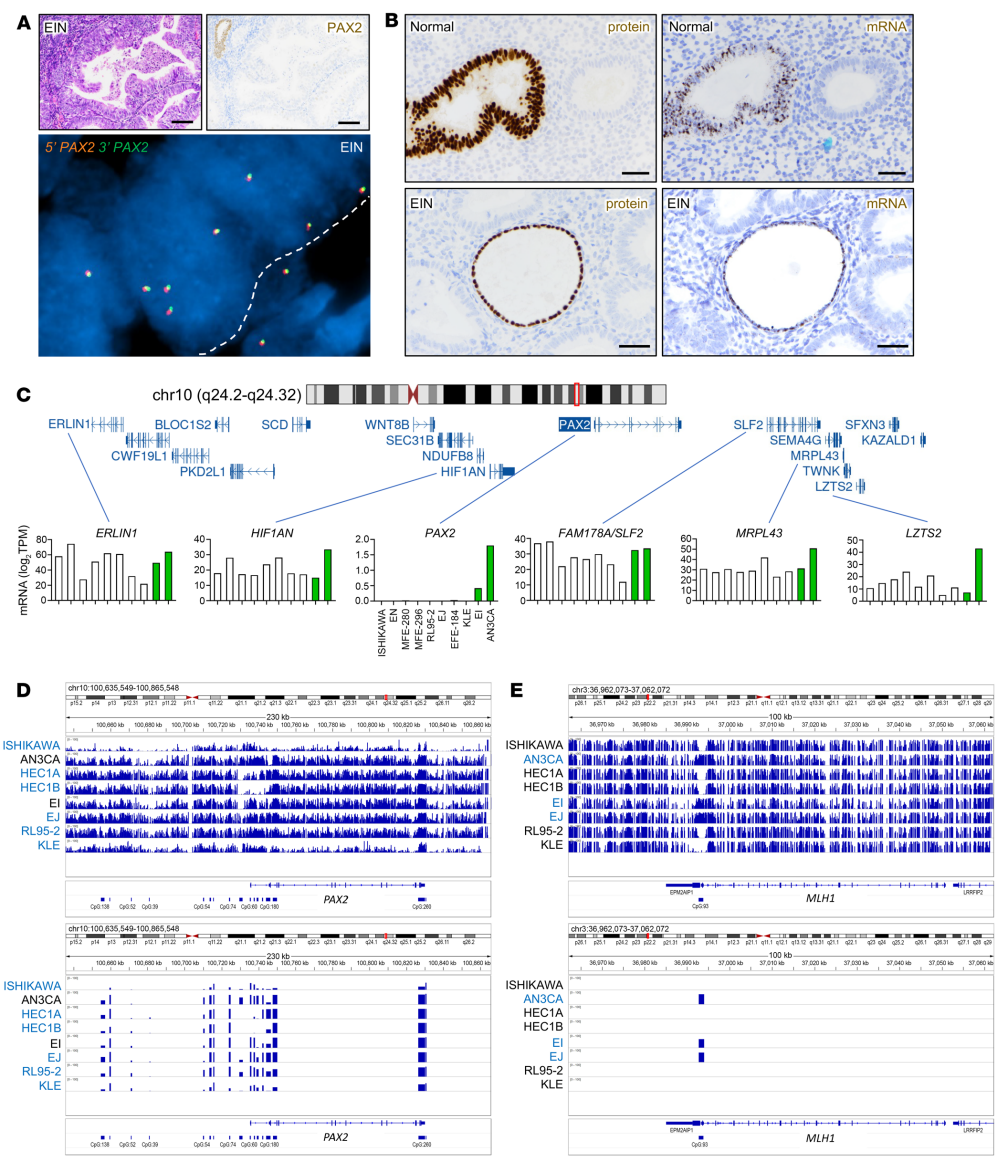
PAX2 protein loss is due to transcriptional silencing specific to *PAX2* locus. (**A**) Top panels: PAX2-deficient EIN. Single gland of residual normal endometrium serves as internal positive control for PAX2 expression. EIN glands show complete loss of PAX2 protein. Scale bars: 200 μm. Bottom panel: break-apart FISH for *PAX2* locus with flanking BAC probes 162 kbp 5′ (Spectrum Orange) and 188 kbp 3′ (Spectrum Green) from *PAX2* gene body in a PAX2-deficient gland. No absent or physically separate orange and green signals are evident. White dashed line demarcates epithelial/stromal boundary. EIN from *n* = 12 patients analyzed with similar results. (**B**) Immunolocalization and RNA ISH of PAX2 loss of expression in serial sections. Top panels: normal human endometrium with single isolated PAX2-deficient gland. Bottom panels: EIN with diffuse PAX2 protein loss. Single entrapped normal (non-neoplastic) gland expressing PAX2 protein (internal positive control). *n* = 6 normal endometria with PAX2-null clones and *n* = 6 EIN with diffuse PAX2 loss were analyzed, with similar results. Scale bars: 50 μm. (**C**) Expression of individual genes adjacent to *PAX2* locus across EC lines per RNA-seq. The *y* axis shows mRNA abundance as log_2_ transcripts/million (TPM). Both PAX2-expressing EC lines are indicated with green bars. (**D** and **E**) Targeted methyl-Seq of *PAX2* (230 kbp) and *MLH1* (100 kbp) coding and flanking genomic regions. CpG islands per UCSC Genome Browser (GRCh37/hg19) shown for both loci (54). Integrated Genomics Viewer shows methylation peaks across both loci. Cell lines highlighted in blue are silenced for the respective locus (*PAX2* or *MLH1*). (**D**) Methyl-seq of *PAX2*. Neither large- nor small-scale methylation events correlated with silencing. (**E**) Methyl-Seq of *MLH1*. Silencing correlated with strong methylation signal in single CpG island known to account for *MLH1* silencing in EC.

*PAX2* point mutations are rare in EC (<1% of cases) and do not explain the high incidence of PAX2 loss (7). Furthermore, the majority of rare EC harboring *PAX2* single-nucleotide coding variants also exhibit ultramutation due to *POLE* mutations, indicating that these *PAX2* variants are “bystanders” due to high mutational burden (21). *PAX3* and *PAX7* chromosomal translocations define childhood rhabdomyosarcomas, raising the possibility that *PAX2* gene rearrangements or deletions might analogously underpin PAX2 expression loss in EC. Break-apart FISH using BAC probes 5′ and 3′ of the gene was conducted in 12 cases of EIN with definitive and complete PAX2 protein loss. In all cases, 5′ and 3′ *PAX2* signals were juxtaposed within the interphase nuclei of EIN epithelial cells, with no loss of either signal. These results ruled out *PAX2* deletions or intrachromosomal rearrangements as the general mechanism accounting for PAX2 inactivation (Figure 2A).

Next, to determine whether PAX2 protein loss in endometrium results from transcriptional mechanisms, we performed *PAX2* RNA ISH (RNAscope) on 6 cases of normal endometria with minute PAX2-deficient clones and 6 cases of EIN. PAX2 immunolocalization was performed on adjacent step sections. Entrapped normal glands served as internal controls. Remarkably, the protein and mRNA loss patterns were superimposable in all 12 cases (Figure 2B). These findings from human tissue specimens establish that (a) PAX2 protein loss occurs at the transcriptional level (gene silencing), and (b) this gene silencing event represents a very early and likely initiating driver event in EC genesis.

### PAX2 silencing is restricted to the PAX2 locus.

The *PAX2* gene (~100 kbp) resides in an approximately 350 kbp gene desert. We sought to determine whether *PAX2* silencing occurred across a larger region and whether neighboring genes were transcriptionally perturbed. RNA-Seq of 10 EC lines, including 2 nonsilenced lines, showed that *PAX2* was the only silenced locus among its genomic neighbors (Figure 2C). Additionally, Western blot analysis of *HIF1AN* (5′ neighbor) showed no downregulation in the *PAX2*-silenced lines (Supplemental Figure 3A). These and the above results establish that *PAX2* is the sole and specific target of a distinct gene-level epigenetic reprogramming event that initiates most EC.

### Abnormal methylation at the PAX2 locus does not explain PAX2 loss.

In EC, hypermethylation of a 5′ *MLH1* CpG island results in locus-specific silencing (5, 22, 23). A previous report suggested that the *PAX2* promoter is normally hypermethylated but becomes unmethylated in EC (19), although this is opposite to *MLH1* and other tumor suppressors subject to promoter hypermethylation. Using the same methylation-specific PCR (MS-PCR) assay, we analyzed non-neoplastic endometria from 40 women. *PAX2* was consistently unmethylated; no specimen exhibited predominant methylation of *PAX2* (Supplemental Figure 3, B and C). Thus, we were unable to reproduce the results of the previous report (19). Another study using this MS-PCR assay also found that *PAX2* was unmethylated in normal endometrium (24).

MS-PCR evaluates the methylation status of only a few bases within a single CpG island. To overcome this limitation, we performed targeted methyl-seq of 230 kbp encompassing *PAX2* in 8 EC lines including the 2 retaining PAX2 expression (Figure 1, G and H). Despite the presence of differentially methylated regions, we did not observe any methylation feature(s) including (a) CpG islands, (b) subregions thereof, or (c) non-CpG regions in the gene body or flanking sequences, which correlated with *PAX2* expression (Figure 2D). In contrast, methyl-seq of a 100 kb *MLH1* region revealed abnormal methylation at only one 5′ CpG island (Figure 2E), where hypermethylation was consistently associated with *MLH1* silencing, as confirmed by Western blotting (Supplemental Figure 3D; see legend for additional details). Thus, neither promoter hypermethylation nor abnormal methylation at the *PAX2* locus is the basis of PAX2 inactivation in EC.

### CRISPR-mediated activation establishes an epigenetic basis for PAX2 silencing and its reversibility.

**Figure 3:**
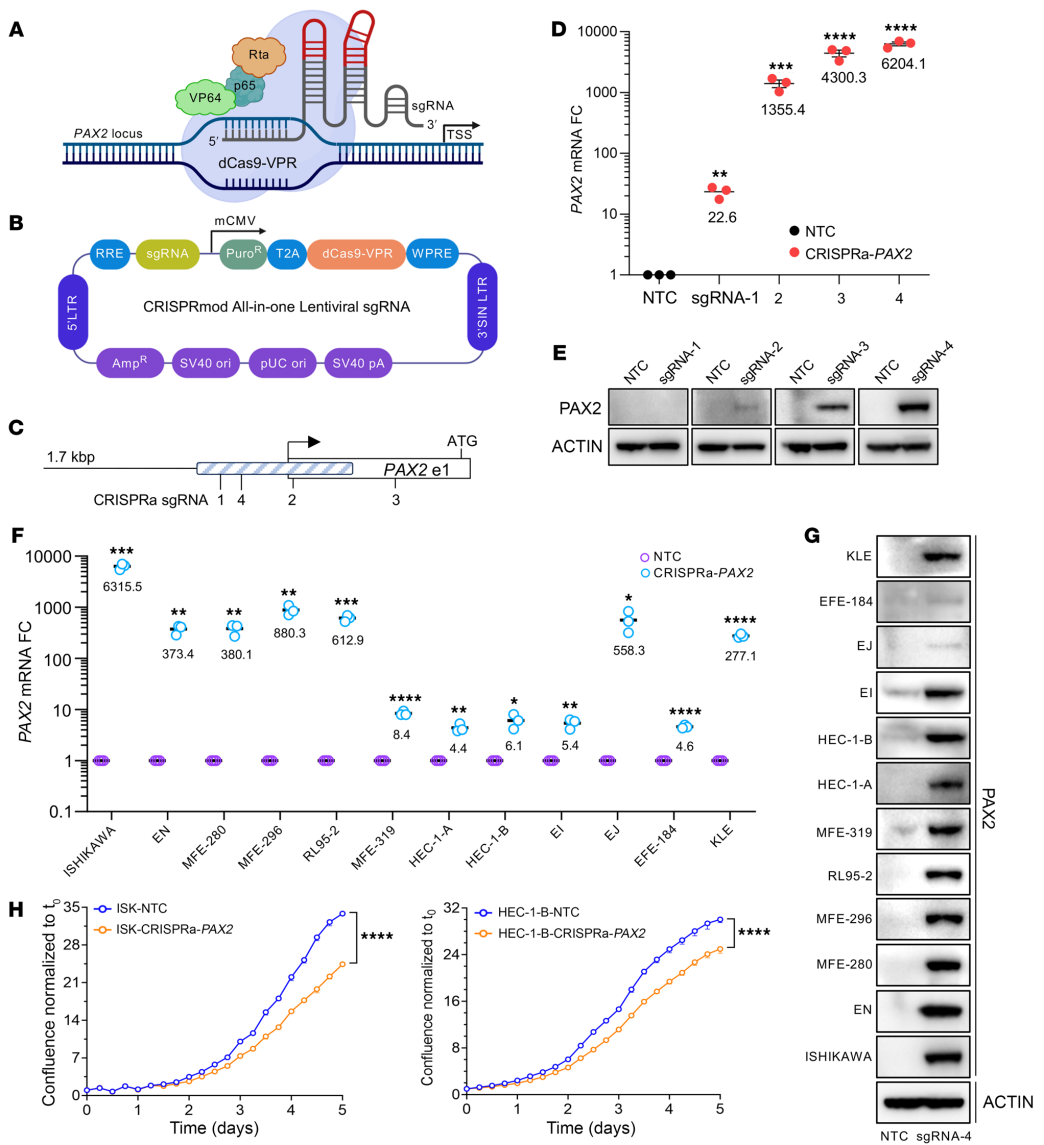
Reversal of *PAX2* silencing by CRISPRa. (**A**) CRISPRa strategy targeting dCas9-VPR to *PAX2* locus with sgRNA (created in BioRender). TSS, transcription start site. (**B**) All-in-one lentiviral construct with sgRNA and dCas9-VPR (created in BioRender). (**C**) Relative positions of 4 sgRNAs to TSS (arrow). Start ATG codon in first exon (unfilled rectangle) is shown relative to 1,598 bp *PAX2* proximal-promoter enhancer region (blue hatched rectangle). The 20 bp sgRNA 4 is 133 bp 3′ of the TSS. (**D**) *PAX2* mRNA expression by qPCR in EC lines following CRISPRa with nontargeting control (NTC) and *PAX2*-specific sgRNAs (*n* = 3, mean ± SEM, multiple *t* tests). FC, fold change. (**E**) Western blot analysis of PAX2 expression in Ishikawa cells after CRISPRa with 4 sgRNAs. (**F**) qPCR of *PAX2* mRNA in Ishikawa cells following CRISPRa with NTC and sgRNA 4 (*n* = 3, mean ± SEM, multiple *t* tests). (**G**) Western analysis of PAX2 expression in EC lines subjected to CRISPRa with sgRNA guide 4. (**H**) Cell proliferation following CRISPRa in Ishikawa (ISK) and HEC-1-B by live-cell imaging (*n* = 3, mean ± SEM, unpaired, 2-tailed *t* test). For all panels, *P < 0.05; **P < 0.01; ***P < 0.001; ****P < 0.0001.

To confirm that another epigenetic mechanism underlies *PAX2* silencing in light of the above unexpected result, we took advantage of CRISPR activation (CRISPRa) using an all-in-one lentiviral vector encoding an endonuclease J-deficient mutant Cas9 (dCas9) fused to the transcriptional activator VP64-p65-Rta (VPR), with a sgRNA targeting the dCas9-VPR transactivator to *PAX2* (Figure 3, A and B) (25). Four sgRNAs targeting *PAX2* were tested, and guide 4, with the strongest *PAX2* induction in Ishikawa, was chosen (Figure 3, C–E). In all 12 EC lines, transduction of the CRISPRa-*PAX2* lentivirus with puromycin selection increased *PAX2* expression per qPCR. Induction levels varied over several logs, from 4–5 times to more than 1,000 times relative to nontargeting control lentivirus (Figure 3F). In all lines, induction of the PAX2 protein was also observed (Figure 3G). Increased *PAX2* expression was most dramatic in *PAX2*-silenced lines such as Ishikawa and MFE-296. Induction was lower in the nonsilenced line EI, as might be expected, but was still significant. Mitotic proliferation assays with Ishikawa and HEC-1-B cells (Figure 3H) demonstrated that *PAX2* CRISPRa resulted in significant mitotic suppression comparable to the enforced expression of *PAX2* cDNA (Supplemental Figure 1E). These findings further demonstrate that the *PAX2* locus is not irreversibly damaged in EC, as would occur with gene deletions/internal rearrangements. In addition, these results constitute a proof of principle that *PAX2* silencing in EC is reversible, with potential therapeutic implications.

### PAX2 silencing is associated with loss of a promoter-proximal active enhancer and gain of facultative heterochromatin features.

**Figure 4:**
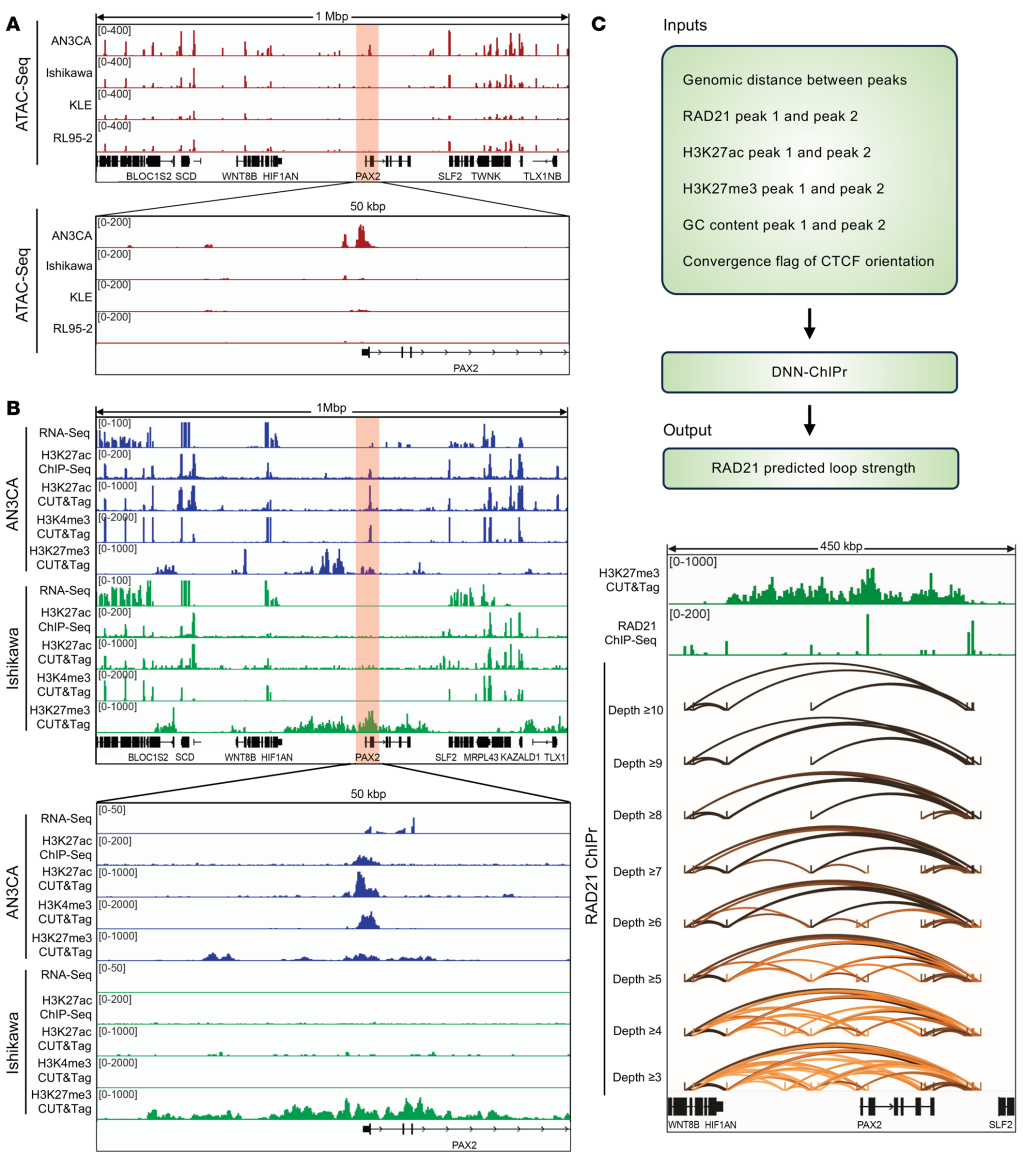
PAX2 silencing is associated with loss of promoter-proximal active enhancer and gain of facultative heterochromatin features. Promoter-proximal enhancer region is chr10:102504680–102506278 (1,598 bp) (GRCh37/hg19). (**A**) ATAC-Seq analysis of *PAX2* locus in AN3CA, Ishikawa, KLE, and RL95-2 cells. AN3CA cells were PAX2+ (nonsilenced), whereas Ishikawa, KLE, and RL95-2 cells were PAX2– (silenced). (**B**) Comprehensive transcriptomic and epigenetic (H3K27ac, H3K4me3, and H3K27me3) profiling of PAX2+ (AN3CA) and PAX2– (Ishikawa) cells. (**C**) Predicted RAD21 ChIA-PET analysis of Ishikawa cells using ChIPr with varying PET interaction strengths, focusing on an H3K27me3-enriched region surrounding *PAX2*. The top panel shows the schematic of the ChIPr pipeline. Interaction strengths are represented by depth values ranging from ≥3 to ≥10.DNN, deep neural networks.

After eliminating small-scale mutations, genomic rearrangements, and abnormal DNA methylation as causes of *PAX2* silencing in EC lines and primary tumors, we investigated alternative chromatin-based epigenetic mechanisms. We analyzed a 1 Mbp region spanning *PAX2* with published assay for transposase-accessible chromatin with high-throughput sequencing (ATAC-Seq) datasets (GSE114964) for PAX2+/nonsilenced (AN3CA) and PAX2–/silenced (Ishikawa, KLE, and RL95-2) EC lines (26). The most striking difference was in the *PAX2* promoter, where an approximately 1.5 kbp region exhibited open chromatin only in the PAX2+ line (Figure 4A). This led us to hypothesize that this active chromatin feature is unique to PAX2+ cells. To profile enhancer activity in this region, we conducted H3K27ac ChIP-Seq on PAX2+ AN3CA versus PAX2– Ishikawa cells, confirming an active enhancer in AN3CA but not Ishikawa cells (Figure 4B). This discovery was independently validated using the H3K27ac CUT&Tag assay. Given the overlap of this enhancer with the *PAX2* promoter, we considered this regulatory element to be a promoter-proximal enhancer that governs *PAX2* transcription.

Consistent with this observation, the *PAX2* promoter was associated with the active promoter mark H3K4me3 in AN3CA but not in Ishikawa cells (Figure 4B). Furthermore, loss of *PAX2* expression in Ishikawa cells was associated with formation of H3K27me3 domains, representing inaccessible chromatin/facultative heterochromatin; this was less pronounced in PAX2+ AN3CA cells and restricted to regions outside the *PAX2* gene body (Figure 4B). These results indicate that *PAX2* transcriptional silencing is associated with loss of open/active chromatin marks and gain of inaccessible chromatin/facultative heterochromatin features. Given that *PAX2* transcriptional silencing is the earliest known initiating event in EC, our results provide insights into the epigenetic basis of this disease.

### Cohesin-mediated 3D genome organization and focal PAX2 silencing in EC.

We further investigated mechanisms underlying *PAX2* silencing and found that it was associated with repressive H3K27me3 marks across the gene desert, but the marks did not spread to neighboring genes, explaining why these *PAX2* neighbors were not transcriptionally affected (Figure 4B). This indicated that the *PAX2* desert may be insulated from neighboring genes through the formation of an insulated gene neighborhood in the context of the 3D genome (27, 28). We hypothesized that the cohesin complex forms an insulated neighborhood via a looping mechanism, restraining the spread of the H3K27me3 domain beyond the desert. To assess this, we analyzed Ishikawa ChIP-Seq data for RAD21 (a cohesin complex component) and observed multiple RAD21 peaks in the *PAX2* gene desert (Figure 4C).

We recently developed Chromatin Interaction Predictor (ChIPr), a machine learning model based on deep neural networks, to predict cohesin-mediated chromatin interaction strengths between any 2 loci (29). Our model uses ChIP-Seq signals for RAD21, H3K27ac, and H3K27me3 as inputs and predicts RAD21 chromatin interaction analysis by paired-end tag sequencing (ChIA-PET) as output. We utilized ChIPr to detect the strength of all combinations of RAD21 loops between RAD21 peaks in the *PAX2* desert. Paired-end tags (PETs) are units used for measuring the interaction strength between a pair of anchor peaks, with more PETs between anchors signifying stronger interactions. We used ChIPr to predict all PETs with a depth of more than 3 between the RAD21 peaks (Figure 4C). Next, we systematically eliminated weak interactions in a stepwise manner by traversing from interactions with PET depths of more than 3 to more than 10. This enabled us to identify the strongest cohesin loops and discovered that the *PAX2* desert is insulated from neighboring genes through the formation of a cohesin-mediated insulated gene neighborhood. Remarkably, the spread of the H3K27me3 repressive domain was perfectly contained within the strong cohesin loops, explaining why the outside genes were not silenced (Figure 4C). Our results indicate that a complex interplay between the 3D genome and the epigenome underlies focal *PAX2* transcriptional silencing in EC models.

We next investigated if the *PAX2* insulated neighborhood is a unique feature of Ishikawa cells or a more generalized feature of all human cells. Analysis of experimental RAD21 ChIA-PET data from 24 human cell types from the ENCODE portal indicated that *PAX2* resides in an insulated neighborhood in all cell lines, making the insulated neighborhood a universal feature of *PAX2* in human cells (Supplemental Figure 4) (30). Given that the loss of many tumor suppressors (*TP53*, *PTEN*, *RB1*, etc.) occurs via focal DNA deletions, *PAX2* transcriptional silencing in the context of cohesin loops is a nongenetic/epigenetic equivalent of focal deletions in cancer.

### Mechanisms underlying PAX2 silencing in EC PDX models and primary EC.

**Figure 5:**
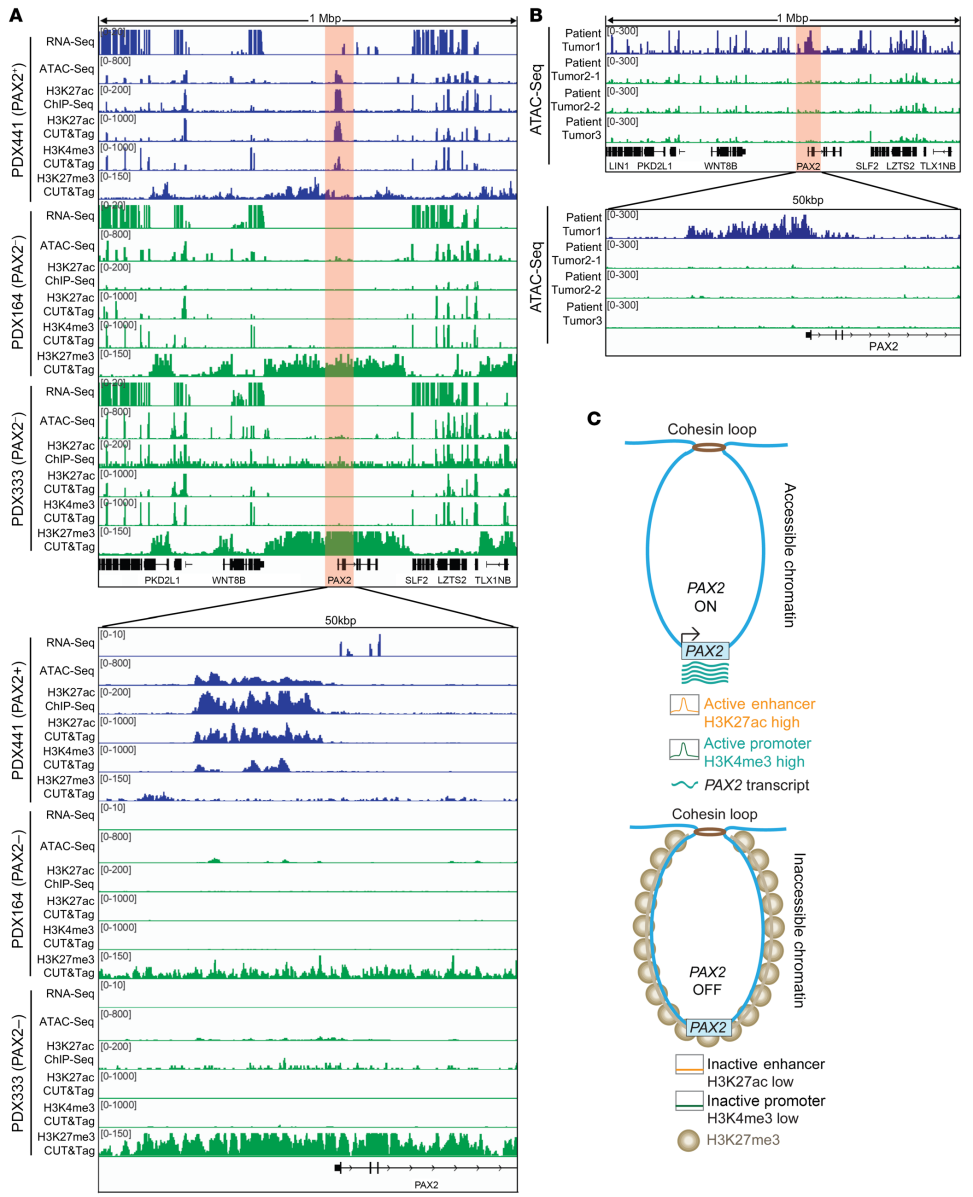
Cohesin-mediated 3D genome organization and focal *PAX2* silencing in EC. (**A**) Comprehensive transcriptomic and epigenetic (ATAC-Seq, H3K27ac, H3K4me3, and H3K27me3) profiling of PAX2+ and PAX2– PDX models of EC. (**B**) ATAC-Seq analysis of *PAX2* locus in primary human EC, including 1 PAX2+ (patient tumor 1) and 2 PAX2– (patient tumors 2 and 3) specimens. Patient tumors 2-1 and 2-2 are technical replicates. (**C**) Pearl necklace model of *PAX2* transcriptional silencing.

Next, we explored the generalizability of these cell line–based discoveries by analyzing EC PDX models. We examined 3 PDXs: 1 PAX2+ (PDX441) and 2 PAX2– (PDX164 and PDX333). We conducted ATAC-Seq and examined the same 1 Mbp spanning *PAX2*. Consistent with our cell line models, the most striking difference was observed for the *PAX2* promoter. In the PAX2+ PDX, this region exhibited open chromatin. Unlike PAX2+ AN3CA cells, where the open chromatin was approximately 1.5 kbp, the open chromatin in the PAX2+ PDX spanned approximately15 kbp (Figure 5A), indicating characteristic differences between the cell lines and PDX models. Enhancer activity was analyzed by profiling H3K27ac using ChIP-seq and CUT&Tag. Both experiments indicated that the open chromatin identified by ATAC-Seq represented a massive enhancer spanning approximately 15 kb, characteristic of a superenhancer, which was unique to the PAX2+ PDX (Figure 5A). Consistent with this observation, the *PAX2* superenhancer exhibited active promoter (H3K4me3) signals in the PAX2+ PDX, but not in the PAX2– PDXs. Conversely, PAX2– PDXs exhibited higher enrichment of the repressive H3K27me3 mark in *PAX2*.

We validated these discoveries by performing ATAC-Seq on primary human tumors. We analyzed 3 patient tumors: 1 PAX2+ (patient tumor 1), and 2 PAX2– (patient tumors 2 and 3) (Figure 5B). For patient tumor 2, there was sufficient material for 2 technical replicates. Consistent with findings in PDX models, the PAX2+ tumor harbored an approximately 15 kbp open chromatin region — indicative of a superenhancer — near the *PAX2* promoter. This feature was absent in the PAX2– tumors. Taken together, these data indicate that the mechanism of *PAX2* transcriptional silencing is remarkably consistent across cell lines, PDX models, and human tumors.

Based on these observations, we propose a “pearl necklace” model for *PAX2* transcriptional silencing (Figure 5C). The loss of *PAX2* expression is associated with the replacement of open/active chromatin features (H3K27ac and H3K4me3) with inaccessible chromatin features (H3K27me3). The spread of H3K27me3 signal resembles a pearl necklace, with its length adjusted by cohesins.

### PAX2 is an oncodevelopmental tumor suppressor regulating endometrial gene expression via control of the enhancer landscape.

**Figure 6:**
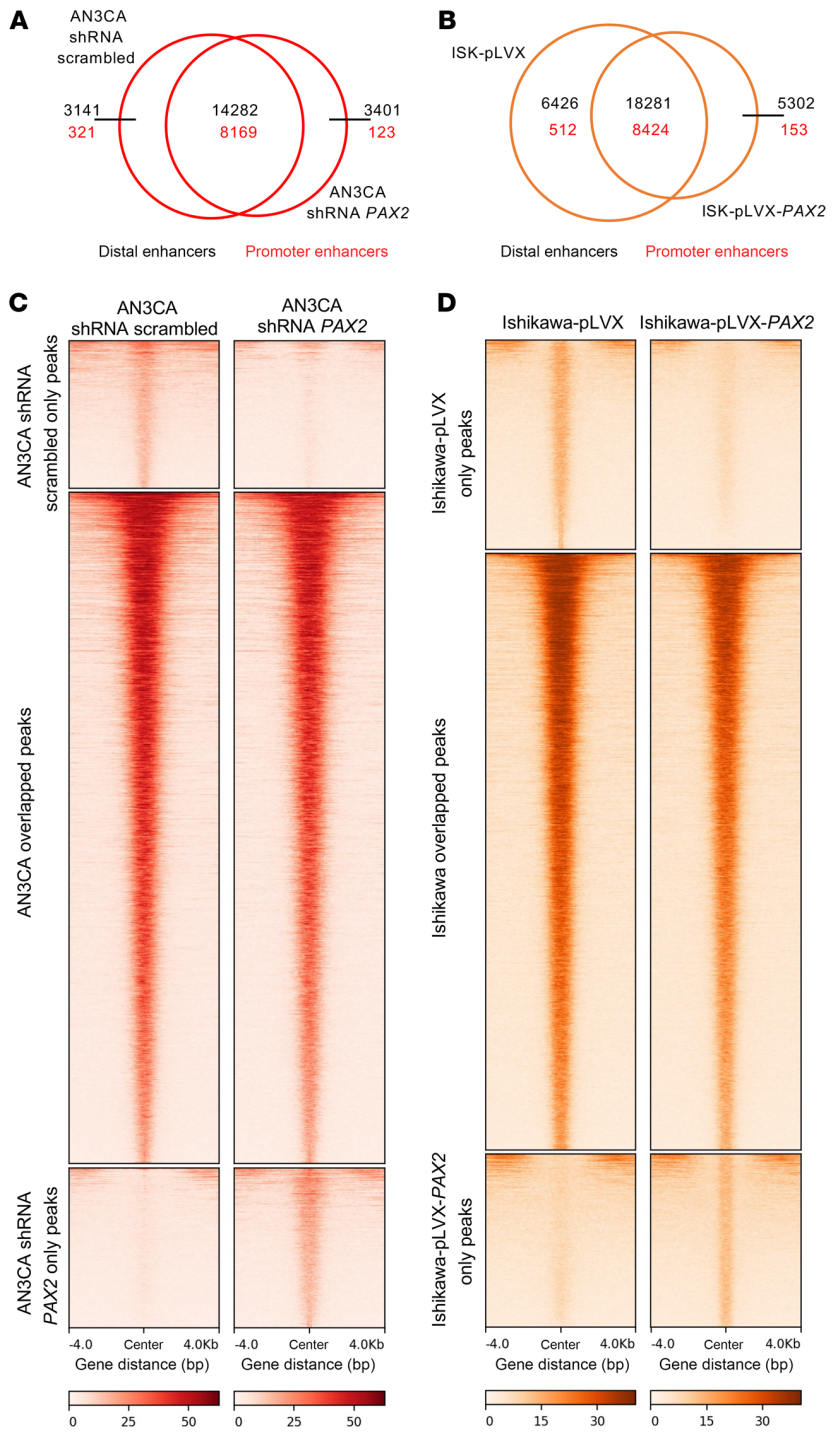
*PAX2* KD and reexpression alter enhancer profiles per H3K27ac ChIP-Seq. (**A** and **B**) Venn diagrams of H3K27ac ChIP-Seq; enhancer peaks in AN3CA (**A**) (scrambled and *PAX2* KD) and Ishikawa cells (**B**) (control and *PAX2* reexpressed) in the promoter and distal regions. (**C**) In AN3CA cells, scrambled shRNA peaks were exclusively identified in scrambled shRNA-treated cells. Overlapping peaks were common between scrambled and *PAX2* KD cells, and shRNA *PAX2* peaks were found in *PAX2* KD cells. (**D**) In Ishikawa cells, empty vector (pLVX) peaks were exclusively present in pLVX-treated cells, overlapping peaks were common between pLVX- and *PAX2*-expressing cells (pLVX-*PAX2*), and *PAX2* reexpression peaks were identified in pLVX-*PAX2* cells.

Prior evidence that *PAX2* is a pioneer TF (31, 32) led us to hypothesize that *PAX2* regulates EC transcriptomes by shaping enhancer activity. We conducted H3K27ac ChIP-Seq to compare enhancer profiles in PAX2+ versus PAX2– (shRNA KD) AN3CA cells and PAX2– versus PAX2+ (reexpressed) Ishikawa cells. In AN3CA, we identified approximately 17.5K H3K27ac peaks with *PAX2* downregulation resulting in both gain and loss of thousands of enhancers (Figure 6, A and C). Similarly, reexpression of *PAX2* in Ishikawa cells also resulted in both gain and loss of thousands of enhancers (Figure 6, B and D). These results indicated that changes in *PAX2* status shaped the chromatin landscape by commissioning and decommissioning enhancers. Notably, these enhancer alterations were predominantly in distal regulatory regions, rather than in gene promoters (Figure 6, A and B). We hypothesized that changes in the activity of these distal enhancers contribute to transcriptomic dysregulation via long-range chromatin interactions.

Reexpression of *PAX2* in Ishikawa cells resulted in hundreds of differentially expressed genes (DEGs) (246 upregulated and 352 downregulated genes; *P* < 0.05, *q* < 0.05, –1 < log_2_fold change > 1), indicating transcriptional reprogramming. Gene Ontology analyses revealed statistically significant enrichment of genes involved in anatomical structure development, developmental processes, and tube development, underscoring PAX2’s role as an oncodevelopmental factor with broad functional impacts (Supplemental Figure 5A). Although PAX2’s expansive impacts on the enhancer landscape and transcriptional reprogramming rationalize its activity as an endometrial tumor suppressor and argue against the overriding significance of individual genes, *PGR* stood out as a potentially significant target, whose expression significantly increased (6.98 times; *P*,*q* < 0.00001) after *PAX2* reexpression in Ishikawa (Supplemental Figure 5B). In ISK-pLVX-*PAX2* cells, progesterone receptors encoded by *PGR* (PR-A/B) were increased at the protein level (2.2 times) (Supplemental Figure 5, B and C). To further validate the functional impact of increased PR-A/B, we treated Ishikawa cells (PAX2+/–) with progesterone and observed modest, albeit significant, suppression of cell proliferation (Supplemental Figure 5D). These findings are consistent with prior results linking PAX2 to the transcriptional regulation of *PGR* (33) and are further explored in mouse models below.

### Mouse model establishes Pax2 as in vivo EC tumor suppressor that synergizes with Pten.

While the above studies provided evidence for a tumor suppressor role of *PAX2* in EC, cell lines have limitations as experimental model systems, including genetic divergence from original tumors and absence of tumor microenvironment plus other host factors (34). To overcome these limitations and test the hypothesis that *PAX2* is an EC tumor suppressor in the most rigorous in vivo genetic system, we explored the biological functions of *Pax2* in genetically engineered mice.

We utilized endometrial epithelium-specific *BAC-Sprr2f-Cre*, which becomes active after sexual maturity at 5 weeks of age (35, 36), and floxed *Pax2* and *Pten* alleles. *Pax2^fl/fl^* mice are viable, whereas *Pax2^del/del^* embryos exhibit renal agenesis, confirming that *Pax2^fl^* yields a null mutation following Cre-mediated gene ablation (37). *BAC-Sprr2f-Cre Pax2^fl/fl^* (*Pax2*), *BAC-Sprr2f-Cre Pten^fl/fl^* (*Pten*), and *BAC-Sprr2f-Cre Pax2^fl/fl^ Pten^fl/fl^* (*Pax2/Pten*) females were generated by breeding. *Pten* was selected because (a) single tumor suppressors usually do not yield overt ECs (35, 36, 38), (b) *PTEN* is the most frequently mutated gene in EC, and (c) *PAX2* silencing and *PTEN* mutations frequently co-occur in EIN and EC (15).

**Figure 7:**
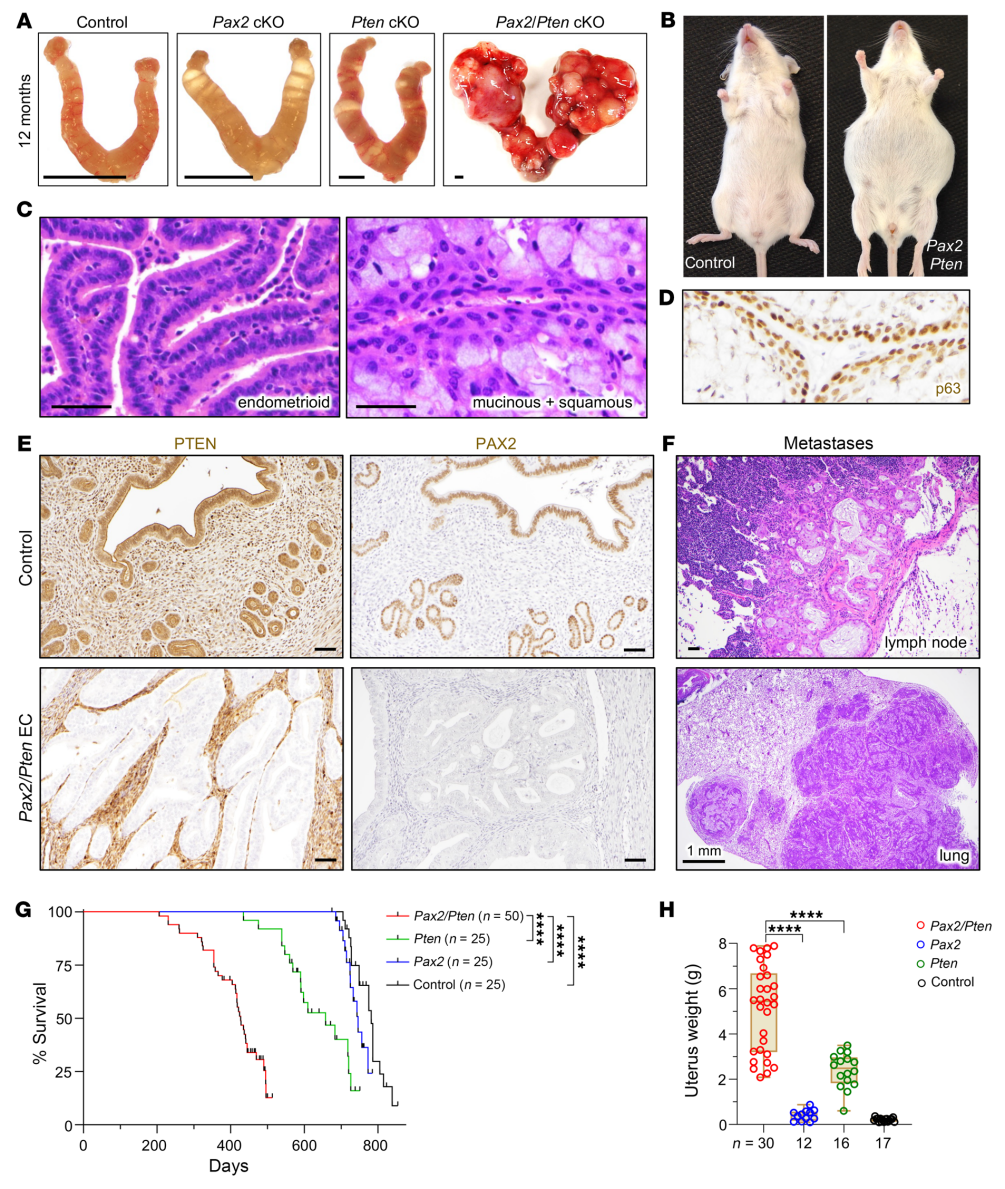
*Pax2* is an EC tumor suppressor synergizing with *Pten* in vivo. (**A**) Uteri at 12 months of age. Scale bars: 2 mm. (**B**) *Pax2/Pten* mouse with distended abdomen due to tumorous uterus and ascites. This phenotype was not observed in single knockouts. (**C**) Distinct EC histotypes in *Pax2/Pten* females, as shown by H&E staining. Scale bars: 50 μm. (**D**) p63 immunostaining in mucinous and squamous EC confirming squamous differentiation. (**E**) PTEN and PAX2 immunostaining confirming endometrial-specific ablation in invasive *Pax2/Pten* EC. Scale bars: 50 μm. (**F**) Distant metastases from *Pax2/Pten* EC, as shown by H&E staining. Scale bars: 50 μm. (**G**) Survival analysis of *Pax2*/*Pten* (*n* = 50), *Pten* (*n* = 25), *Pax2* (*n* = 25), and littermate control (*n* = 25) mice. ****P < 0.0001 per log-rank test. (**H**) Uterine weights at necropsy; the *x* axis shows number of animals per genotype. ****P < 0.0001, 1-way ANOVA, Tukey’s multiple-comparison test.

Consistent with prior studies, endometrium-specific *Pten* inactivation resulted in EIN, with only some *Pten* mice developing lethal adenocarcinomas with very long latency (36, 38–40). In contrast, *Pax2/Pten* females exhibited a striking and lethal phenotype with early mortality due to EC (Figure 7, A and B). Tumors exhibited 2 discrete histotypes: endometrioid (16/30 mice, 53.3%), endometrioid mucinous with squamous differentiation (confirmed by p63; 1/30, 3.3%), or an admixture of both (13/30, 43.3%) (Figure 7, C and D). Mucinous and/or squamous differentiation are common features of human EC. Thus, while 2 distinct tumor histotypes were observed in *Pax2/Pten* EC, often together, both fell within the spectrum of human EC. Immunolocalization confirmed loss of PTEN and PAX2 in malignant epithelial cells (Figure 7E). *Pax2/Pten* ECs were highly invasive, often into the abdominal cavity, with frequent metastases in 30 mice subjected to complete necropsy, including ovary (60%), kidney (16.7%), liver (10%), pancreas (16.7%), spleen (10%), and intestine (33.3%), with distant metastases in lymph nodes (6.7%) and lung (16.7%) (Figure 7F).

*Pax2* mice showed a minor decrease in survival, with only a subset displaying early signs of invasive EC; however, this was not statistically significant. In contrast, *Pax2*/*Pten* mice had significantly shorter median survival than littermate controls or single-knockout mice (Figure 7G). Uterine weights also confirmed striking cooperativity. While *Pax2* mice had normal uterine weights and *Pten* mice had increased uterine weights due to longer uterine horns rather than invasive cancers (41), *Pax2*/*Pten* mice had far higher uterine weights reflecting overt tumor burden (Figure 7H). In summary, these results provide formal genetic evidence that *Pax2* is an in vivo EC tumor suppressor that synergizes with *Pten*, establishing mice as a useful model for additional investigations into the biology of *Pax2* in EC.

### Single-cell RNA-Seq reveals PAX2-null population and validates PGR as a PAX2 target.

**Figure 8:**
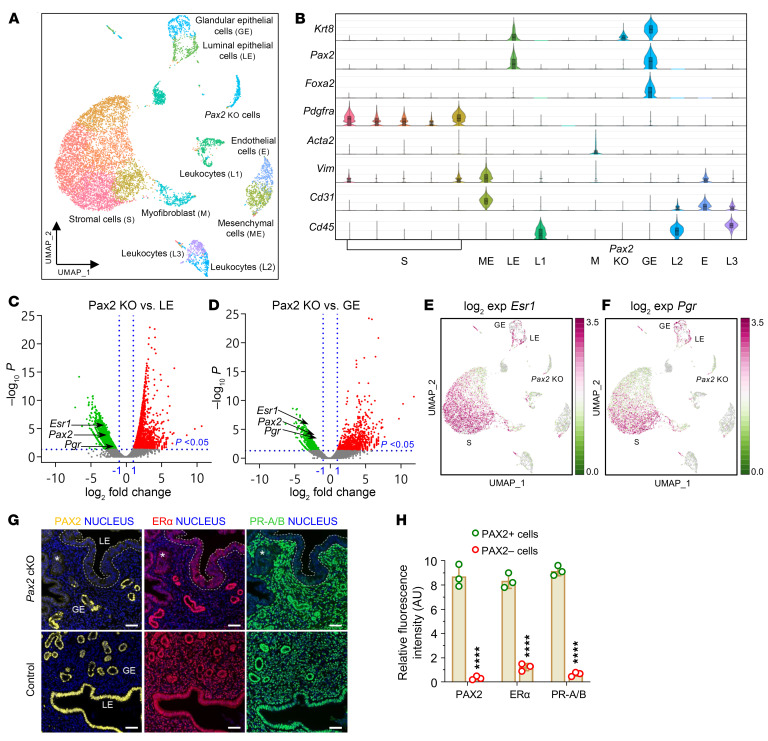
scRNA-seq reveals that inactivation of *Pax2* loss correlates with reduction of *Esr1* and *Pgr* expression in mouse endometrium. Studies were performed with *Pax2*-mosaic uteri at 8 weeks of age. (**A**) Uniform Manifold Approximation and Projection (UMAP) visualization showing cells from *Pax2* mouse uterus (*n* = 2) clustered into 15 distinct subpopulations based on established lineage markers. (**B**) Stacked violin plots showing expression of gene signatures associated with known uterine cell types, facilitating identification of lineages within clusters. (**C** and **D**) Volcano plots showing DEGs (*P* < 0.05) in *Pax2*-KO cluster compared with both (**C**) luminal epithelial (LE) and (**D**) glandular epithelial (GE) cell clusters. Vertical dotted lines represent log_2_ fold change threshold of ±1, while the horizontal dotted line represents a *P* value threshold of 0.05. Selected genes are shown. (**E** and **F**) UMAP plots of *Esr1* (**E**) and *Pgr* (**F**). (**G**) Immunofluorescence staining for PAX2, Erα (*Esr1*), and PR-A/B (*Pgr*) in control and *Pax2* mouse uterine adjacent sections (*n* = 3). GE, glandular epithelium; LE, luminal epithelium. Scale bars: 50 μm. (**H**) Comparison of relative fluorescence intensities of PAX2, ERα, and PR-A/B between PAX2+ and PAX2– cells. Data are shown as mean ± SEM (*n* = 3); ****P < 0.0001, multiple 2-tailed *t* tests.

Our EC analyses indicated that *PAX2* regulates *PGR*. Previous studies have shown that among established EC lines, only Ishikawa cells express ER-α and PR-A/B (38, 42). To explore whether loss of *PAX2* is associated with a reduction in *PGR* in vivo, we performed single-cell RNA sequencing (scRNA-Seq) of *Pax2*-mosaic uteri at 8 weeks (see next section and Supplemental Figure 7A for explanation of mosaic system). Uniform Manifold Approximation and Projection plots revealed diverse uterine populations, including stromal cells, together with glandular and luminal epithelial cells, among other cell types (Figure 8A). Violin plots for selected informative genes are shown in Figure 8B; for example, *Foxa2* distinguishes luminal from glandular epithelial cells (38), whereas *Krt8* marks both luminal and glandular epithelial cells. The identification of a distinctive *Pax2*-null epithelial cell cluster permitted DEG analyses relative to PAX2+ luminal and glandular epithelium, both of which identified *Pgr1* as underexpressed in the PAX2– epithelial population (log_2_fold change –1.74, *P* = 0.02 for glandular epithelium), consistent with the EC line data implicating *Pgr* as one of many *Pax2* targets (Figure 8, C–F). Immunofluorescence of tissue sections from mosaic uteri showed that ER-α and PR-A/B were underexpressed in PAX2– cells relative to their PAX2+ neighbors, whereas controls had uniform expression levels of both factors, confirming that *Pax2* regulates their expression (Figure 8, G and H).

### Organoids reveal synergistic growth phenotypes in 3D culture.

Epithelial organoids were isolated from control, *Pax2*, *Pten*, and *Pax2/Pten* mice at 12–16 weeks, before the onset of malignancy at 30 weeks. At this time point, organoids should reflect phenotypes associated with a specific engineered mutation(s) rather than the acquisition of additional mutations. Remarkably, *Pax2* inactivation alone produced a distinct phenotype of larger organoids with intraluminal growth, resulting in solid organoids versus controls, which formed single-layer, hollow structures (Supplemental Figure 6, A and B). This was more clearly observed in serial sections of organoids obtained by confocal *Z*-stack imaging (Supplemental Figure 6C). *Pten* inactivation resulted in even larger organoids with hollow lumina, as described previously (36, 38). In contrast, *Pax2*/*Pten* organoids were significantly (*P* < 0.0001 at each timepoint) larger than the single knockouts (Supplemental Figure 6, A–C). Moreover, these double-knockout organoids also exhibited aberrant growth into lumina, suggestive of EMT phenotypes (36). These synergistic growth patterns were evident as accelerated growth and increased cell numbers in 3D culture (Supplemental Figure 6, D and E).

The *Pax2* single-knockout organoid phenotype is remarkable, as it shows that Pax2 loss alone confers cellular phenotypes that favor growth. To further explore this, we took advantage of mosaic patterns of *Pax2* ablation resulting from subtotal *BAC-Sprr2f-Cre*–mediated recombination in young females, leading to coexistence of mutant PAX2– and PAX2+ cells within glands (Supplemental Figure 7, A and B). Initially, organoids from *Pax2* mice at 8 weeks of age contained PAX2+ cells (~35%–40%) (Supplemental Figure 7, C and D). However, after serial passaging, PAX2+ cells rapidly declined and disappeared by the third passage (Supplemental Figure 7, C and D). Since the culture medium lacked estradiol (required for *BAC-Sprr2f-Cre* expression), ex vivo loss of PAX2 expression was not likely due to sustained Cre activity (36). Control organoids exhibited 100% PAX2 expression throughout serial passages (Supplemental Figure 7, C and D), as confirmed by confocal *Z*-stack imaging (Supplemental Figure 7G). The loss of PAX2+ cells was further validated using *Pax2* qPCR (Supplemental Figure 7, E and F). These results demonstrated a significant competitive growth advantage of PAX2– over PAX2+ cells, further rationalizing the emergence of PAX2-null clones in human endometrium.

Taken together, these studies highlight the following key findings: (a) *Pax2* negatively regulates endometrial cell proliferation in vivo, (b) PAX2-deficient cells outcompete their normal counterparts, and (c) potent synergism between *Pax2* and *Pten* significantly affects cell proliferation and tumor phenotypes. These observations also rationalize the observed loss of PAX2 in EC and its frequent co-occurrence with *PTEN* mutations/PTEN protein loss.

## Discussion

Cancer is driven by cell-heritable alterations that promote abnormal cell proliferation and insensitivity to physiological control mechanisms. Most documented cancer-driving events are DNA-level mutations, reflecting the ease with which such mutations have been reliably identified at the genomic level through DNA sequencing. One insight from these studies has been the identification of recurring oncogenic mutations in genes controlling chromatin architecture and gene expression, establishing deregulation of epigenetic control mechanisms as a hallmark of cancer (11, 43). Mutations in chromatin regulatory factors have broad and pleiotropic effects that alter transcription at the genome-wide level, a phenomenon that should be distinguished from nonmutational epigenetic reprogramming events targeting single loci.

DNA methylation at CpG dinucleotides was the first epigenetic mark identified. CpG dinucleotides are abundant near transcriptional start sites of housekeeping genes, and such promoter CpG islands are almost always unmethylated. While CpG methylation is considered an epigenetic mark, it involves chemical modification of DNA, which distinguishes it from other types of epigenetic alterations based on histone codes. Many tumor suppressor loci, especially those that are also broadly expressed housekeeping genes, harbor CpG promoter islands (44). Through yet unknown mechanisms, promoter CpG islands of some tumor suppressor loci such as *MLH1* become hypermethylated, leading to transcriptional gene silencing through recruitment of repressor proteins, chromatin remodeling, or blocking TF binding. Such hypermethylation events appear to occur in a single cell that then gains a clonal advantage, with promoter hypermethylation status stably maintained by DNA methyltransferases during cell division and tumor growth (11).

PAX2 is expressed in only a small number of tissues and cell types, including the parathyroid and genitourinary tract (kidney, seminal vesicle, and uterus), where it serves critical functions in organogenesis and development. Unlike ECs, renal cell carcinomas retain PAX2 expression (45). PAX8 is also highly expressed in endometrium but does not undergo loss in EC (46), making PAX2 silencing a distinctive signature lesion of the endometrium and, as far as is known, unique among the PAX family as a tumor suppressor in the female reproductive tract or elsewhere. PAX2’s status as a tissue-specific oncodevelopmental factor is consistent with our results that promoter hypermethylation is not the underlying mechanism for PAX2 silencing, given that most tumor suppressor loci subject to CpG island hypermethylation are broadly expressed housekeeping genes.

Our mouse models establish *PAX2* as an in vivo endometrial tumor suppressor synergizing with *PTEN*. Together with our demonstration by RNA ISH that gene silencing underlies PAX2 loss in minute clones in human endometrium, and the widespread inactivation of PAX2 in 80% of EIN, this study points to *PAX2* silencing as the principal driver event initiating many if not most ECs. However, *PAX2* silencing is not the only initiating event nor is it an obligate one, as evident from its retained expression in 20% of ECs (15). How *PAX2* silenced versus nonsilenced ECs differ biologically, or if some other molecular events serve as surrogates for *PAX2* silencing, is unknown. Focal PTEN loss in single glands also occurs in some normal endometria, although at lower incidence than PAX2. Per prior reports, such minute PTEN-null clones (which likely harbor biallelic *PTEN* mutations) (47) express PAX2, while conversely, minute PAX2-null clones express PTEN (14). Yet, many EIN are deficient for both PTEN and PAX2 (15). This suggests that while the order of inactivation is flexible, there is selection for inactivation of both during EC progression. Mutations in other genes resulting in PI3K pathway hyperactivity, such as *PIK3CA* or *PIK3R1*, are also common in ECs (7). Such mutations can functionally substitute for *PTEN* inactivation and may synergize with *PAX2* silencing.

*PAX2* transcriptional silencing in EC is comparable to *ERG* transcriptional upregulation in prostatic adenocarcinoma, which is strikingly similar to EC. Aging is the primary risk factor for EC and prostate cancer. Both exhibit precancerous histological counterparts, EIN and prostatic intraepithelial neoplasia (PIN), driven by transcriptomic dysregulation. Upregulation of ETS TFs (primarily ERG) and downregulation of *PAX2* are observed in approximately 20% of PIN and approximately 80% of EIN lesions, respectively, and facilitate transition to carcinoma (48). In the prostate, upregulation of ETS TFs and PI3K pathway activation cooperatively drive the transition from PIN to prostate adenocarcinoma (49, 50). Likewise, in the context of the endometrium, we now show in our mouse models that inactivation of *Pax2* together with PI3K pathway activation (via *Pten*, the most frequently mutated gene in EC) cooperatively drives the transition from EIN to EC.

Our study shows that a combination of (a) loss of open/active chromatin marks and (b) gain of inaccessible chromatin/facultative heterochromatin features in a framework dictated by cohesin-mediated 3D genomic architecture underlies focal *PAX2* silencing. Although developed to explain *PAX2* transcriptional silencing, we speculate that our pearl necklace model could be generalized to other cancer drivers. Our discoveries open new questions for the field: What are the upstream triggers for the loss and gain of mutually exclusive H3K27ac and H3K27me3 signals, respectively? Given the intricate association of the endometrium with temporal (long- and short-term) changes in hormonal signaling, we speculate that depletion of master TFs in the *PAX2* enhancer may lead to H3K27ac signal loss. This may be stochastic or linked to a normal aging process. We also note that when *PAX2* is expressed, the locus is partially bivalent in terms of H3K27ac and H3K27me3 signals. In particular, H3K27me3 signal is not completely lost (Figure 4B and Figure 5A). Therefore, transient loss of H3K27ac signals can result in the gain of H3K27me3 signals. However, as H3K27me3 contributes to compact and inaccessible chromatin, reestablishment of the H3K27ac signal may become less likely from a biochemical standpoint. This epigenetic switch from H3K27ac to H3K27me3 is likely to undergo positive selection, as we have shown that *PAX2* is a tumor suppressor. The cohesin loops serve as guard rails to prevent this biochemical event from spilling over to neighboring genes. Another question is, how does a stochastic chromatin remodeling event silence *PAX2* in a single cell, which then expands into a minute PAX2-deficient clone? Silencing one copy of *PAX2* may provide a small competitive advantage. If so, one *PAX2* copy may be silenced initially, followed by a second stochastic event involving the other allele to completely silence *PAX2*. Alternatively, both alleles may be silenced simultaneously via an unknown mechanism.

Not surprisingly, it has proven difficult to pharmacologically reconstitute the activity of missing or inactive tumor suppressor proteins, making classical tumor suppressors such as TP53 and PTEN ineffective targets. Nonetheless, observations from this study support PAX2 as an actionable target. First, PAX2 loss defines the large majority of primary and metastatic EC, making PAX2-based strategies of broad potential clinical impact. Second, although heterogeneity is a major factor limiting treatment efficacy, PAX2 loss is an initiating event and usually a molecular feature across ECs. Third, our CRISPRa studies showed that PAX2 was reactivable in all EC lines, and this had phenotypic consequences, confirming that the locus was not irreversibly damaged in EC. Fourth, the CRISPRa results represent a proof of principle that PAX2 could be reactivated pharmacologically. Small-molecule inhibitors of diverse epigenetic modifier enzymes may lead to the reactivation of PAX2 (10), and novel agents could be identified through systematic chemical screening for *PAX2* reexpression (5).

In summary, this study establishes a specific *PAX2* epigenetic reprogramming event as a highly recurring cancer-initiating mechanism in EC. We have developed a number of resources, including cell lines, PDXs, epigenomic datasets, and a genetically engineered mouse model, that we employed to answer fundamental questions and are well suited for future investigations to explore further details about *PAX2*’s function as a tumor suppressor or its interactions with PI3K/PTEN and other cancer-causing pathways. These findings have diverse implications for the diagnosis and clinical management of EC, a common but underestimated malignancy in women.

## Methods

Additional methods are described in Supplemental Methods.

### Sex as a biological variable.

Our study exclusively examined female mice because the disease modeled is only relevant in females.

### Nomenclature.

Per standard HUGO nomenclature, in this manuscript, *Pax2* is a mouse gene or mRNA, *PAX2* is a human gene or mRNA, and PAX2 is a mouse or human protein.

### Human tissues.

For *PAX2* methylation PCR analysis, immunohistochemistry, and in situ hybridization, human endometrial tissue sections (both normal and cancerous) were obtained from FFPE tissue blocks (UT Southwestern Clinical Pathology Laboratories). Normal archival FFPE endometrial specimens for age studies were retrospectively identified from biopsies for workup of abnormal uterine bleeding and where the histologic diagnosis was proliferative endometrium (the preovulatory phase of the menstrual cycle when the endometrium is mitotically active). Abnormal endometrial architecture and history of endometrial neoplasia were exclusion criteria. Tissue microarray sections (US Biomax) from samples from patients with EC were utilized for PAX2 immunohistochemistry and determination of H-scores in grade 1–3 EC.

### Mouse strains and survival analysis.

Endometrial epithelium-specific *Pax2* and/or *Pten* homozygous conditional knockout mice were generated by breeding mice harboring floxed *Pax2* (maintained on a 129/Sv × C57BL/6J mixed background) (37) and *Pten* (*Pten^tm1Hwu^*/J, stock 004597) (51) alleles with *BAC-Sprr2f-Cre* mice (maintained on an FVB background, but now available in a pure C57/B6 background through The Jackson Laboratory as [B6(FVB)-Tg (*Sprr2f*-*cre*)2DCas/J; stock 037052] (36). Sibling progeny inheriting either a single floxed *Pax2* or *Pten* allele but lacking the *BAC-Sprr2f-Cre* transgene were used as controls. The mice were housed in a pathogen-free animal facility in individually ventilated cages and fed ad libitum on a standard chow diet under a 12:12 hour light–dark cycle. Survival analyses were conducted on both experimental and sibling control mice selected at the time of weaning.

### Cell lines.

Human EC cell lines Ishikawa (Sigma 99040201), HEC-1-A (ATCC HTB-112), HEC-1-B (ATCC HTB-113), AN3CA (ATCC HTB-111), RL95-2 (ATCC CRL-1671), KLE (ATCC CRL-1622), EN (DSMZ ACC 564), MFE-319 (DSMZ ACC 423), MFE-280 (DSMZ ACC 410), MFE-296 (DSMZ ACC 419), EFE-184 (DSMZ ACC 230), and EI and EJ (obtained from M Takayama, Tokyo Medical University, Japan) (52, 53) were cultured in MEM/RPMI/DMEM-F12 medium (Gibco) per ATCC recommendations supplemented with 10% heat-inactivated FBS (Sigma) and antibiotics (50 U/mL penicillin and 50 mg/L streptomycin; Thermo Fisher Scientific) at 37°C in a humidified atmosphere with 5% CO_2_. The cell lines were routinely tested to confirm their *Mycoplasma*-negative status using a Mycosensor PCR assay kit (Agilent Genomics; 302108).

### Statistics.

For all experiments, data are presented as the mean ± SEM unless otherwise stated, and statistical analyses were performed using GraphPad Prism (v.10.0.0). Fisher’s exact test was used to determine the *P* value for PAX2 loss between young and aged women. PAX2 H-scores among different grades of patients were determined using Dunnett’s multiple-comparison test. Statistical significance between 2 groups or multiple groups was assessed by unpaired, 2-tailed Student’s *t* test and 1-way ANOVA, respectively. Kaplan-Meier analysis and the log-rank test were used to determine the *P* value between survival curves. Each in vitro experiment was repeated thrice, and the in vivo experiments were repeated twice. Statistical significance was set at *P* < 0.05.

### Study approval.

Primary tumor samples from patients with EC were obtained immediately after hysterectomy at the UT Southwestern Medical Center or the University of North Carolina School of Medicine. The samples were collected with written informed consent from all the patients under research protocols approved by the respective Institutional Review Boards at UT Southwestern Medical Center and the University of North Carolina School of Medicine.

All animal procedures and experiments were conducted in accordance with the guidelines and regulations approved by the UT Southwestern Medical Center IACUC.

### Data availability.

Values for all data points in graphs are reported in the Supporting Data Values file. The data supporting the findings of this study, including methyl-Seq, RNA-Seq, scRNA-Seq, ATAC-Seq, ChIP-Seq, and CUT&Tag-Seq datasets, were deposited in the National Center for Biotechnology Information’s Gene Expression Omnibus database and are accessible through accession numbers GSE275208, GSE275345, GSE275320, GSE275221, GSE275222, and GSE275223. ATAC-Seq data for patient tumors are available through accession number GSE294692.

## Author contributions

DHC, RSM, SSS, and SGR conceived the study. SSS, SGR, YG, SL, AA, XZ, AK, and CX curated data and performed formal analysis and visualization. DHC and RSM acquired funding. SSS, SGR, ICC, PK, and SR performed investigations and designed methods. DHC, RSM, and CX contributed to project administration and supervised the study. SR, RRB, VLBJ, ABG, JL, EL, and AK provided resources. DHC, RSM, SSS, and SGR wrote the original draft and reviewed and edited the article.

## Supplementary Material

Supplemental data

Unedited blot and gel images

Supporting data values

## Version 1 08/15/2025

Electronic publication
