# Supplementary figures and images for: A distinct mechanism of epigenetic reprogramming silences PAX2 and initiates endometrial carcinogenesis
Source: J Clin Invest. 2025 Aug 15;135(16):e190989. doi: 10.1172/JCI190989 (PMC12352900; doi:10.1172/JCI190989)

Fig 1G.

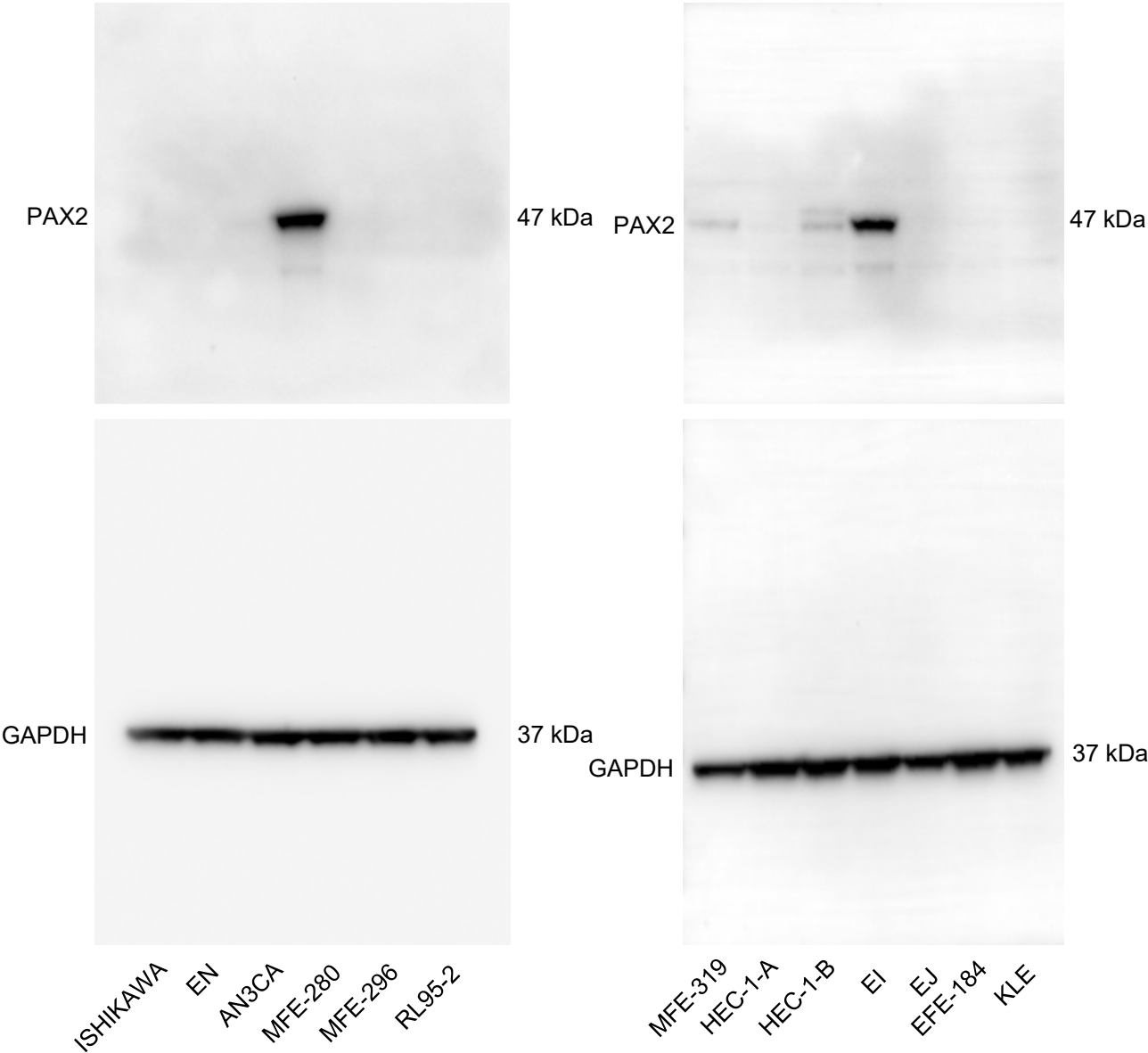

**Fig 3E.**

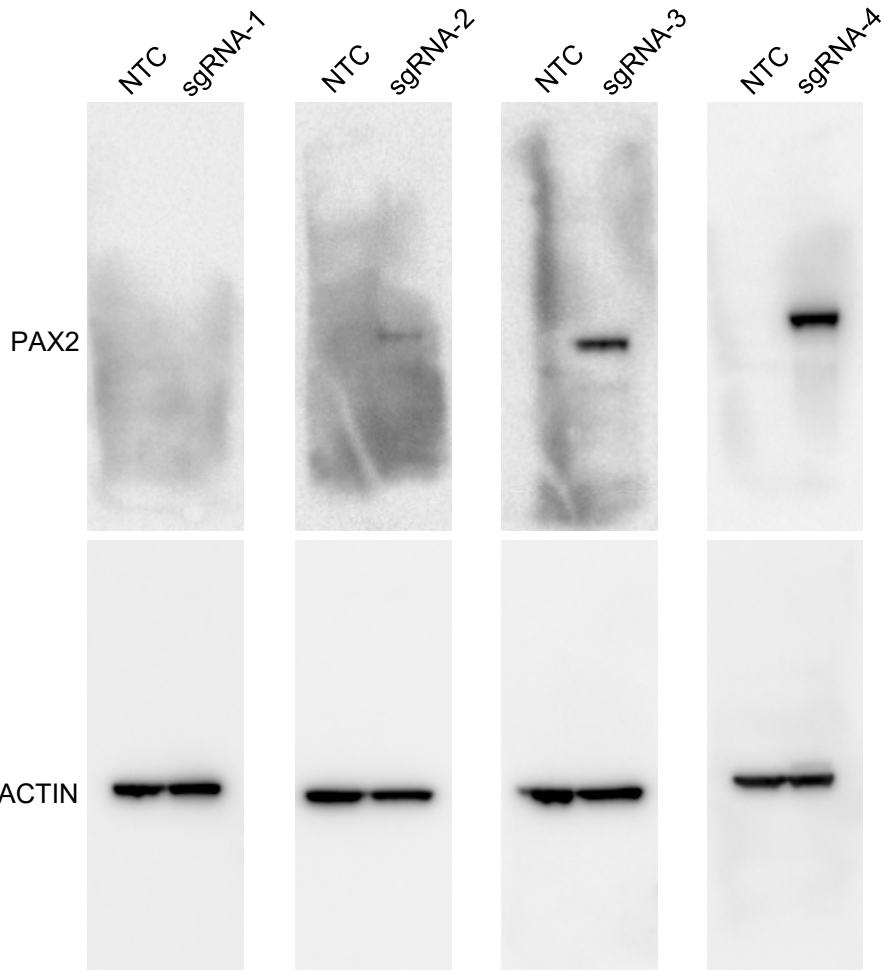

**Fig 3G.**

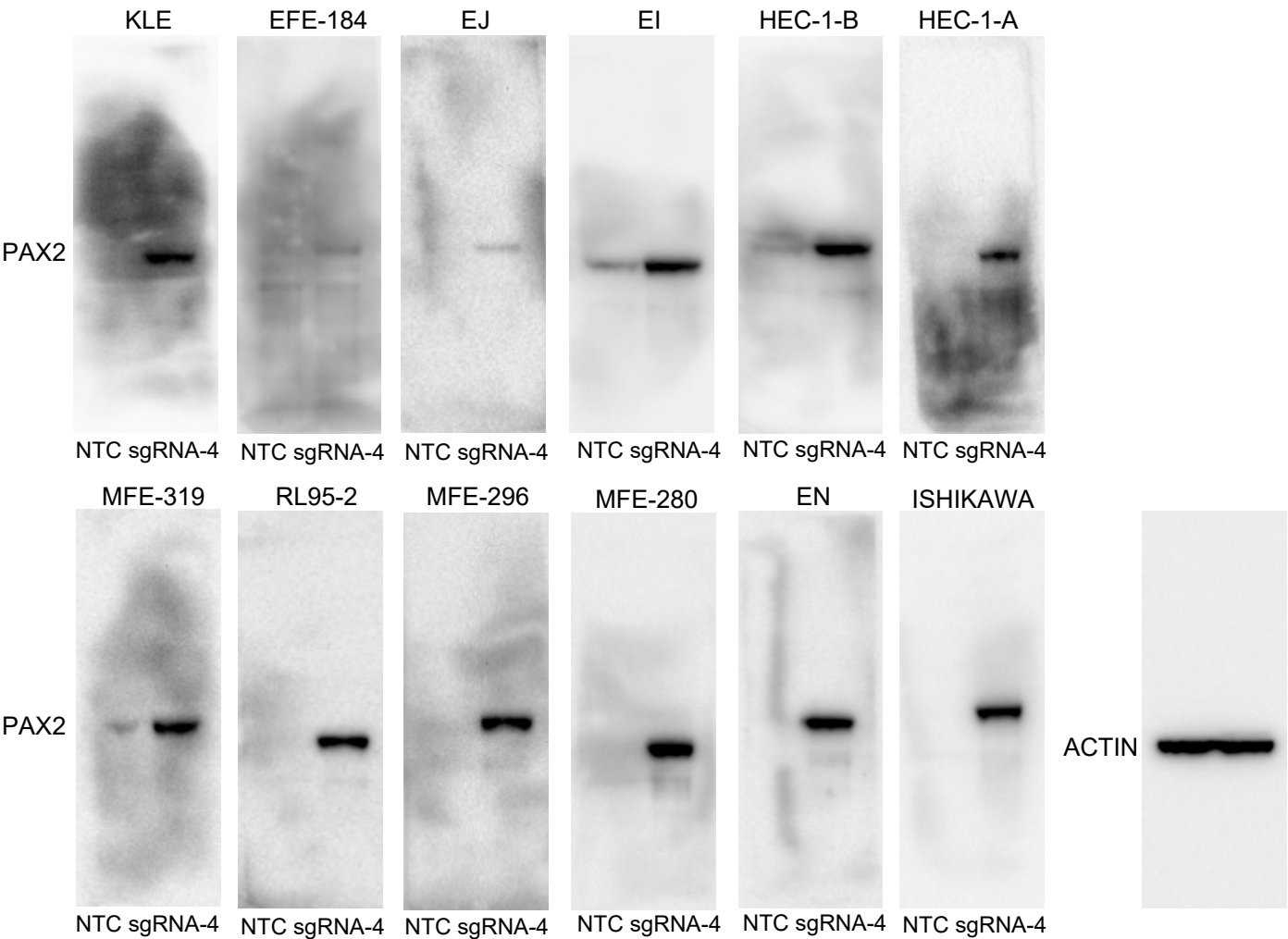

**Fig S1B.**

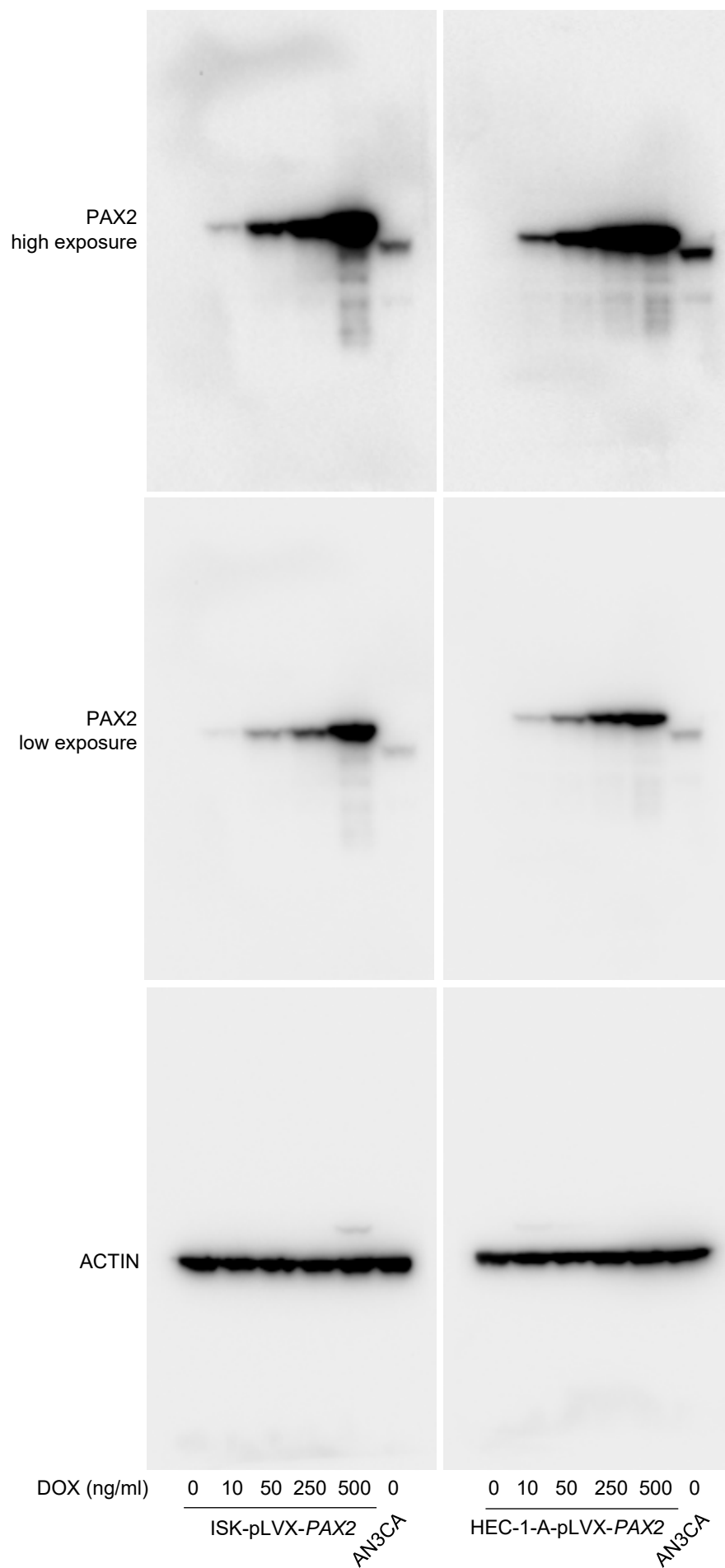

**Fig S1C.**

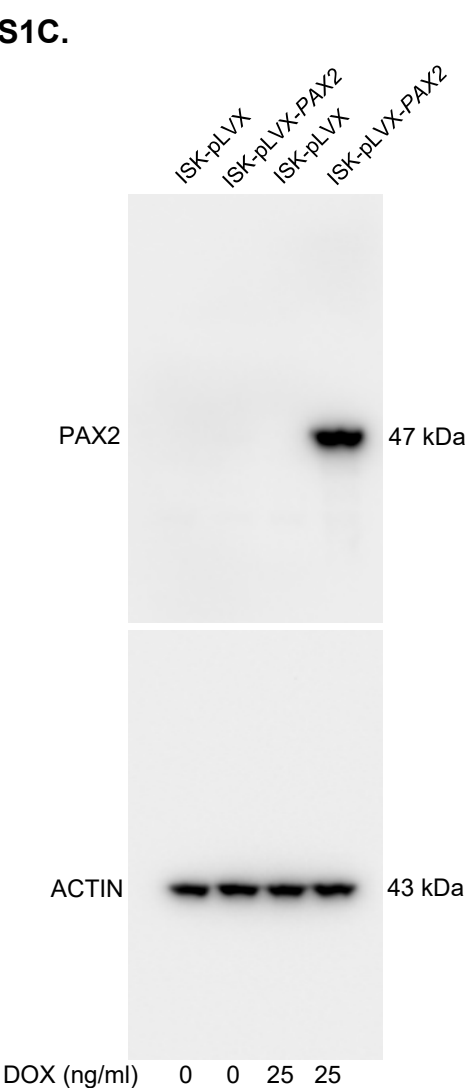

**Fig S1M.**

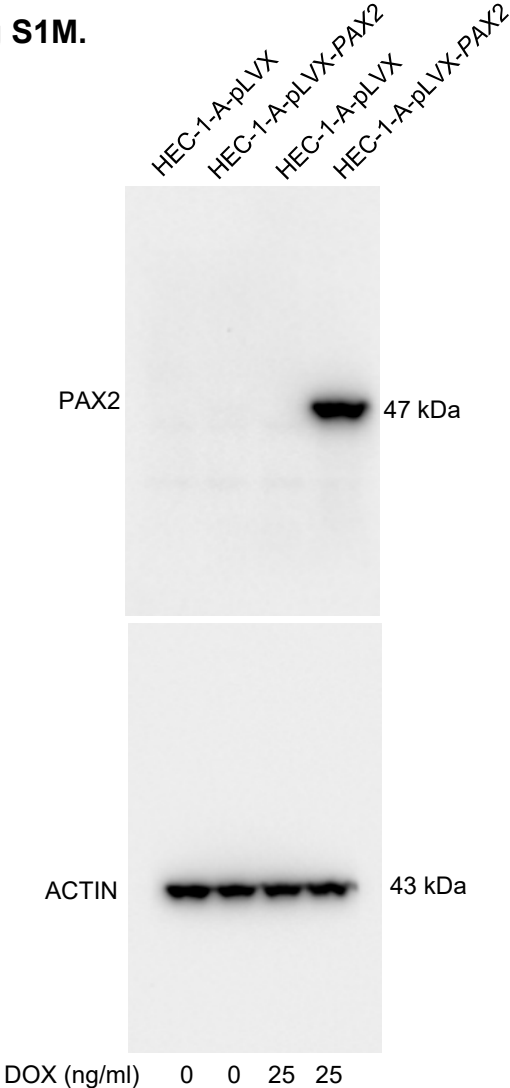

**Fig S1K.**

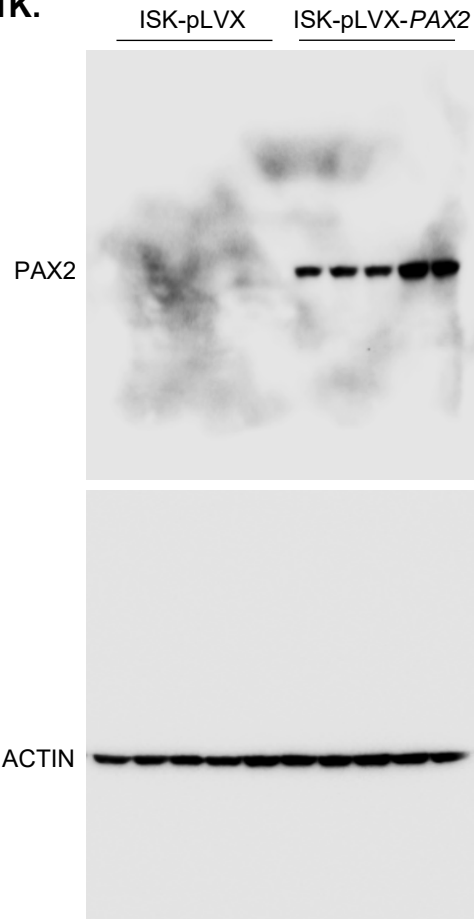

**Fig S1U.**

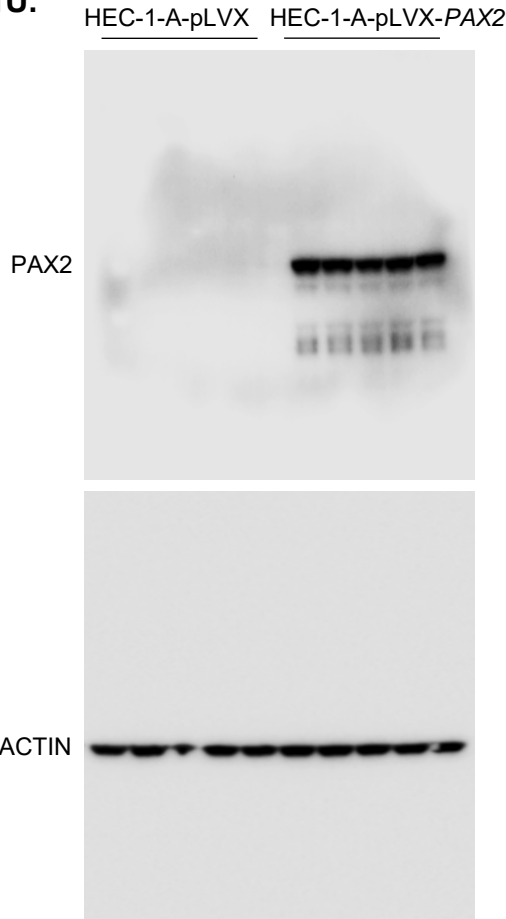

**Fig S2B.**

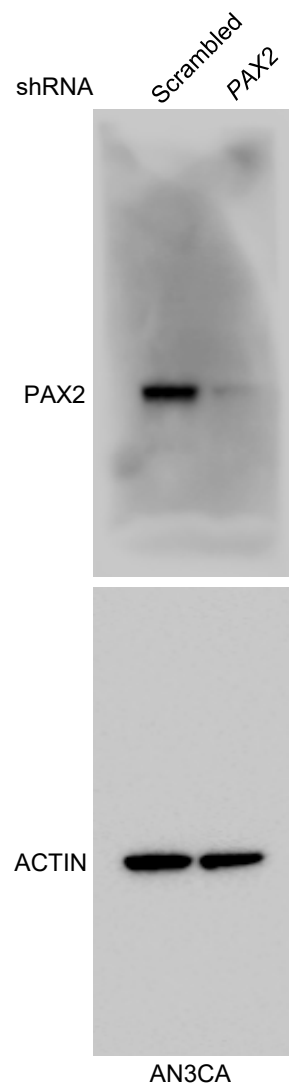

**Fig S2L.**

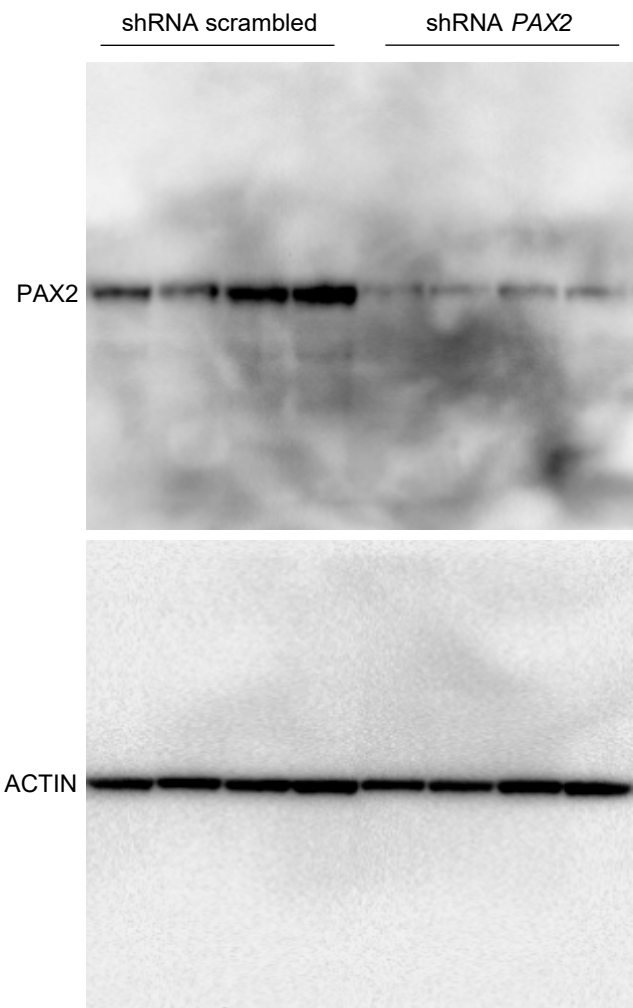

**Fig S3A.**

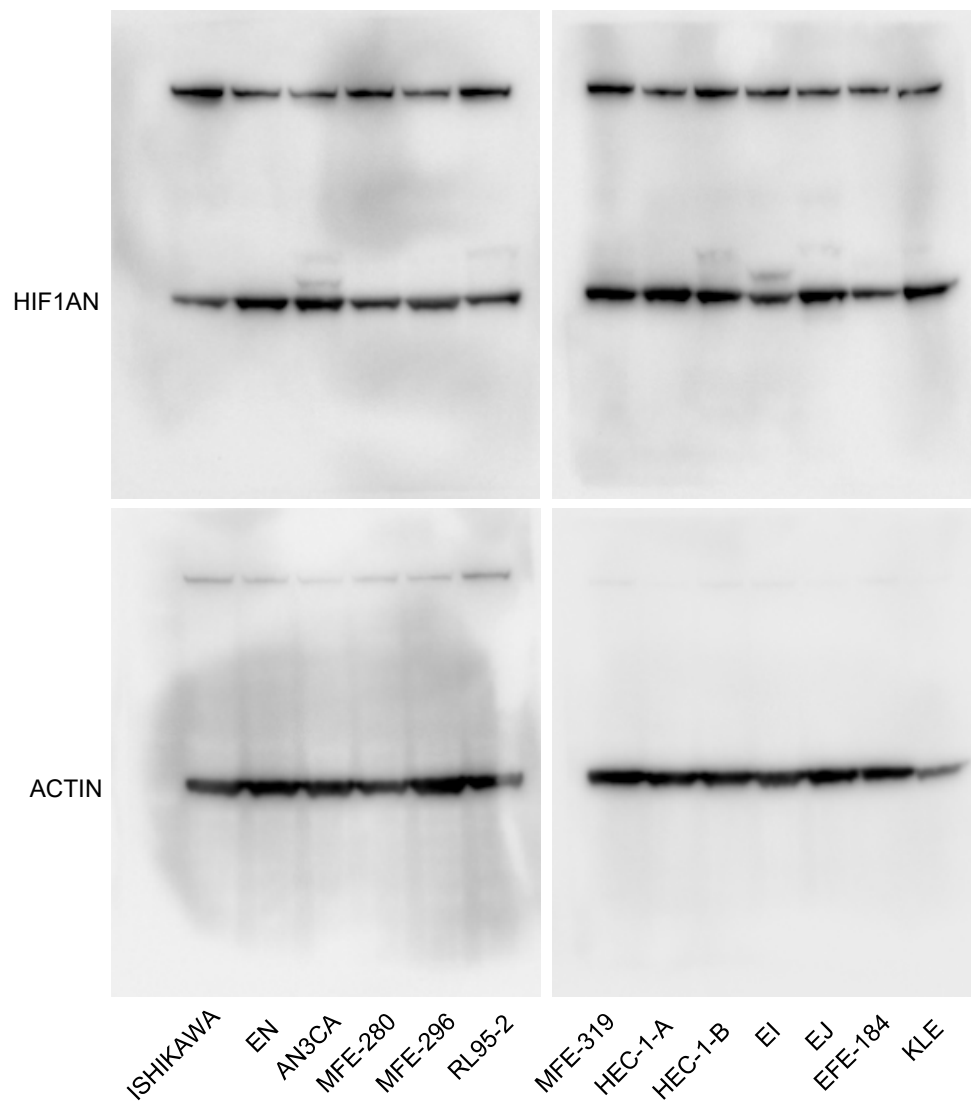

**Fig S3D.**

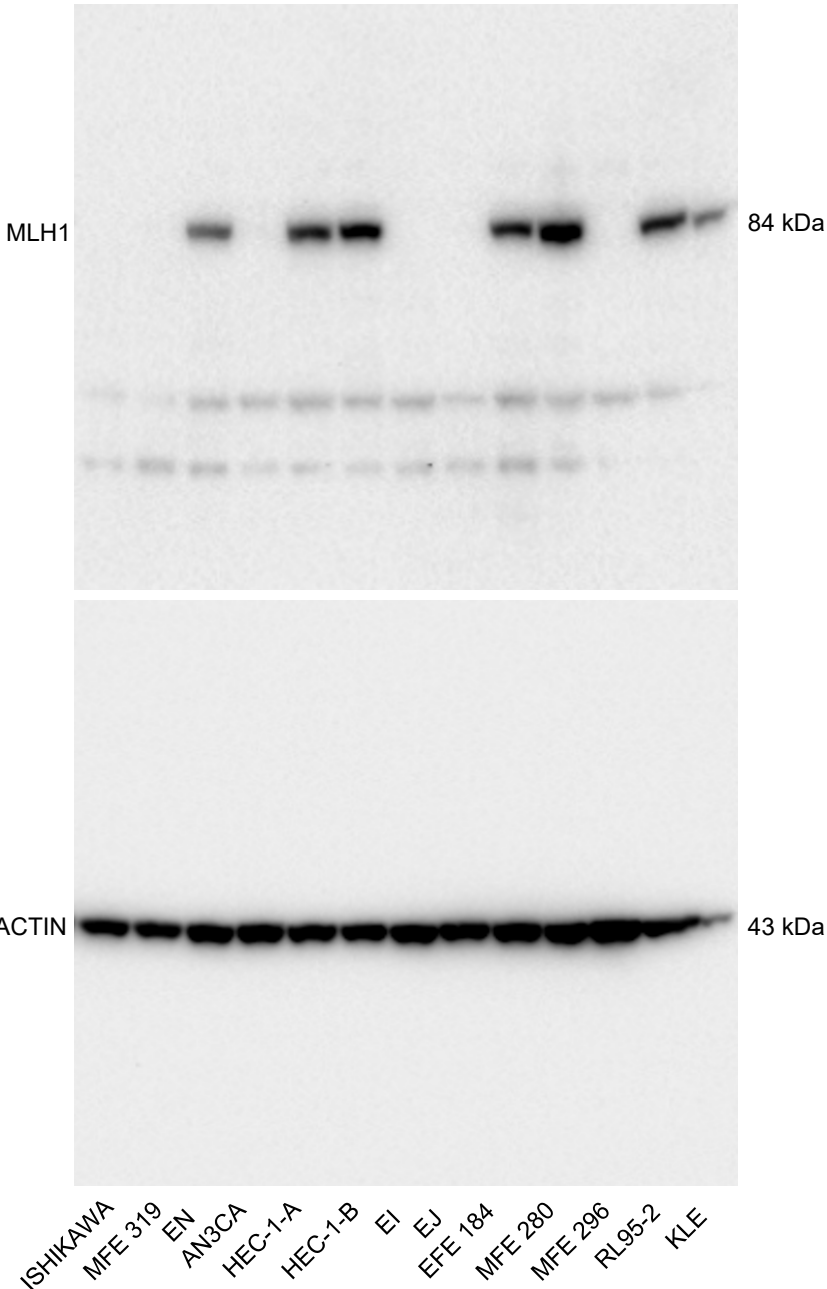

**Fig S5C.**

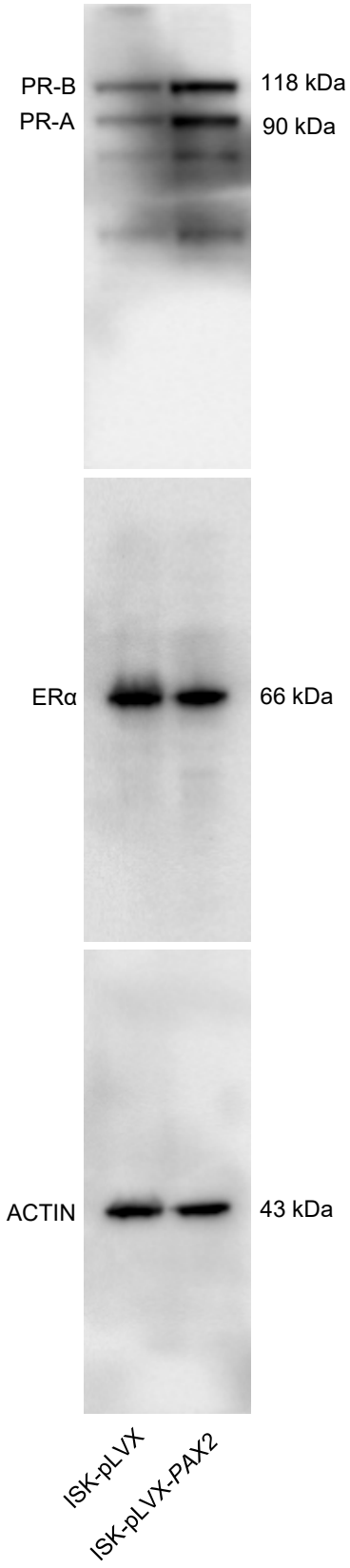

Supplement: Unedited blot and gel images [file jci-135-190989-s065.pdf]
